# Relative Influence of Pd(II) and Pt(II) Metal Centers on the Strength of Interactions Between Planar Species

**DOI:** 10.1002/asia.71018

**Published:** 2026-10-18

**Authors:** Jess L. Algar, Rosemary J. Goodwin, Dan Preston

**Affiliations:** ^1^ Research School of Chemistry Australian National University Canberra Australia

**Keywords:** metal–metal interactions, palladium, platinum, π interactions

## Abstract

Palladium(II) or platinum(II) are often incorporated into metallo‐supramolecular architectures as their square‐planar geometry and relative accessibility make them attractive targets. The square‐planar cationic panels where palladium(II) or platinum(II) are bound within these species frequently participate in interactions which enable host–guest chemistry or controlled conformation. We here investigate whether there could be a difference between the strength of interactions established by Pd(II) or Pt(II)‐containing species. Analogous Pd(II) and Pt(II) model complexes were synthesized, and their behavior compared against aromatic and platinate guests. Predominantly through ^1^H NMR techniques, we have established that Pt(II) can allow for π interactions with an aromatic guest roughly twice the strength of their Pd(II) counterparts, where binding is strong. Similarly, the interactions between the Pt(II) model complex with a platinate guest, were stronger than those of the Pd(II) model complex, although notably weak in both cases.

## Graphical abstract

Pt(II)‐containing square planar complexes can associate with organic and inorganic guests more strongly than their Pd(II) analogues.

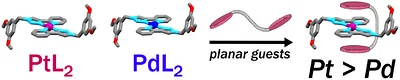

## 1 Introduction

The self‐assembly of supramolecular architectures is guided by a wide range of supramolecular interactions. Dynamic covalent chemistry or metal coordination, for example, are highly directional, relatively less reversible, and in turn crucial to controlling the basic identity and connectivity of components in a self‐assembly. In contrast, π interactions and solvent effects are more diffuse, highly reversible, and in turn more often contribute to conformation. Nonetheless, a combination of these interactions, irrespective of reversibility, is often essential in directing the assembly of a particular target.

In nature, π interactions are frequently observed between amino acids with aromatic side chains, often essential in stabilizing the tertiary structure of proteins [1]. Within artificial systems, they have been shown to affect connectivity [2], conformation [3, 4, 5, 6], and function particularly regarding guest recognition [7]. π interactions can exist in many forms, including between electron‐rich aromatics; electron‐rich aromatics and cations; aromatics with inverted electron density; or aromatics in which electron density has been altered due to metal coordination. Given the wide variety of contexts where these types of interactions have been observed, traditional terms like π–π stacking have rightly been questioned as of late, with more generalized terminology being proposed such as “electrostatics, dispersion, desolvation, induction and exchange‐repulsion” (EDDIE) [8]. For simplicity in describing this work, we choose to use the term π interactions to refer to the favorable, largely electrostatic interactions between π systems. We make a concerted effort to avoid terms like π–π stacking, which we regard as a more useful descriptor in a crystallographic sense.

Similar to π interactions, metal–metal interactions have been observed, often crystallographically, manifesting as shortened metal–metal distances generally between heavier metal ions with d^8^ or d^10^ configurations. Metal–metal interactions most notably account for the difference in color of Magnus’ salts [9], [Pt(NH_3_)_4_][PtCl_4_], between the solid‐state and solution phase [10, 11, 12, 13]. Since then, recognition of platinate guests by a variety of metal‐containing receptors such as metallo‐tweezers and lantern‐shaped cages has been identified [14, 15, 16, 17, 18, 19, 20, 21, 22, 23]; demonstrating these interactions can also exist in the solution phase. However, it has been noted that these interactions are not well understood, relatively weak, and often effectively nonexistent in particular solvents [24].

Within metallo‐supramolecular chemistry, square planar metal ions such as palladium(II) and platinum(II) are popular choices. It is worth noting that when combined with monodentate ligands, these metal ions have proved excellent candidates in the formation of many impressive self‐assemblies. In the present report, however, we instead focus solely on their combination with chelating ligands to give planar complexes by virtue of the square‐planar metal ions. The inter‐ or intra‐molecular interactions that can form from the resulting planar cationic panels often dictate the host–guest (HG) chemistry of the system or control its conformation. With growing interest and capability in altering or combining distinct metal ions within a system [25, 26, 27, 28, 29, 30], it is becoming essential to thoroughly understand not only the interactions themselves, but also how they influence one another and their combined effects.

Molecular recognition of guests via π interactions or metal–metal interactions has been studied, mostly in the field of metallo‐tweezers [31]. A range of metals, including platinum(II) [14, 15, 18, 32, 33, 34, 35, 36, 37], gold(I) [23, 38, 39, 40], gold(III) [41, 42], and palladium(II) [14, 37] bound within the backbone of metallo‐tweezers can act to create an electron‐deficient cleft suitable for aromatic guest binding through π interactions or directly bind platinate guests through metal–metal interactions. Significant theoretical and crystallographic work has investigated these interactions [43, 44, 45, 46, 47]. However, we were interested in whether the identity of the metal ion of the host can significantly affect the strength of interactions with aromatic or platinate guests, specifically comparing palladium(II) and platinum(II) model complexes experimentally. Palladium(II) and platinum(II) have similar ionic radii and in turn similar M─N bond lengths due to the lanthanide contraction effect. Consequently, the main differences between the two metal ions are instead related to their electron density and electropositivity. We propose that the larger electron density of platinum(II) could enhance the strength of these interactions given their electrostatic basis. On the other hand, platinum(II) is less electropositive, which would suggest the opposite behavior could dominate, or perhaps these effects would cancel out to give no observable differences.

Previous work by Bosnich [37] observed slightly improved molecular recognition of 9‐methylanthracene (*K*_11 _= 650 M^−1^ to *K*_11_ = 800 M^−1^ in CD_3_CN at 27°C) and a platinate guest (*K*_stab_ = 12,000 M^−1^ to *K*_stab_ = 51,000 M^−1^ in CD_3_CN at 70°C) within platinum(II) based molecular tweezers over their palladium(II) counterparts. Considering these values and particularly the temperature of the platinate guest measurements, it is difficult to conclude from these examples alone if the metal ion identity does affect the strength of association. Furthermore, these association constants were derived from metallo‐tweezers which feature two metal centers per guest and are not necessarily representative of many hosts where the binding of a guest may occur on either side of the host, such that two guests may bind per metal center. Resnati, Frontera, and coworkers [44] have computationally investigated the energetic differences of interactions between aromatic rings and group 10 metal centers. Their work revealed stronger interactions form from platinum(II) relative to palladium(II). We aimed to explore this further experimentally.

In this study, we report a simple model system of the form ML_2_, where M is either palladium(II) or platinum(II) and detail a comparison between the model complexes’ ability to recognize electron‐rich aromatic guests, to assess π interactions, and a platinate guest, to assess metal–metal interactions. The knowledge from this study should be able to be applied to larger systems and hopefully predict and/or rationalize the behavior of these platinum(II) and palladium(II) containing self‐assembled species.

## 2 Results and Discussion

### 2.1 Ligand and Guest Design

A simple triazole‐pyridyl‐based ligand (**AD‐Bz**) bearing a dimethoxybenzene sidearm was first synthesized (Figures S1 and S2). The ligand was named **AD‐Bz**, where AD represents the hydrogen bond acceptor (A) and donor (D) capability of the binding site, in line with previously used naming conventions [2, 4, 48], and Bz denotes the dimethoxybenzene sidearm. The calculated electrostatic potential (ESP) surfaces of the complexes (Figure 1f [49, 50]) reveal the relatively electron‐poor nature of the metal‐coordinated face, making these complexes suitable candidates for molecular recognition of electron‐rich aromatic guests. **AD‐Bz** and [Pd(CH_3_CN)_4_](BF_4_)_2_ were combined in a 2:1 ratio at room temperature and a white precipitate isolated following trituration. To synthesize the Pt(II) analogue, [Pt(DMSO)_2_Cl_2_] was combined with AgBF_4_ in a 1:2 ratio and heated at 50°C in the absence of light for 20 min. The solution was centrifuged to remove the precipitate and then combined with 2.5 eq. **AD‐Bz** and heated at 50°C for 4 h. Following trituration, another white precipitate was isolated.

**FIGURE 1:**
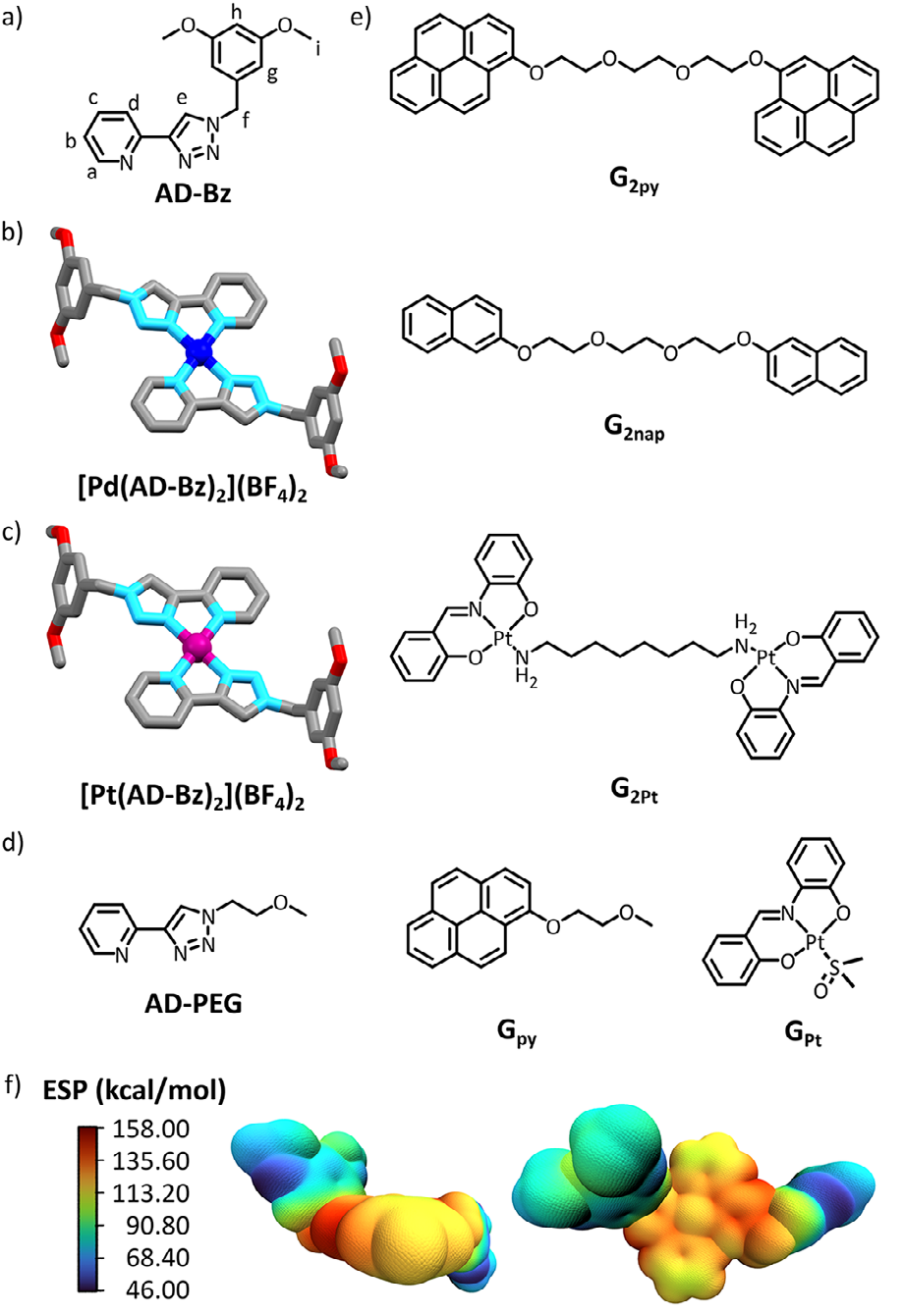
Chemical representations of (a) **AD‐Bz**, (b) X‐ray crystal structure of **[Pd(AD‐Bz)_2_](BF_4_)_2_**, (c) X‐ray crystal structure of **[Pt(AD‐Bz)_2_](BF_4_)_2_**, (d) AD‐PEG (used only for crystallizations), (e) the chosen guests, **G_2py_**, **G_2nap_**, and **G_2Pt_** as well as variants **G_py_** and **G_Pt_**, and (f) ESP [49, 50] of **[Pt(AD‐Bz)_2_](BF_4_)_2_** from a side (left) and top view (right).

Both precipitates were dissolved separately in [D_6_]DMSO, and a single set of peaks was observed within each ^1^H NMR spectrum, having shifted downfield relative to the free ligand, indicative of metal coordination (Figures S16 and S20). ^1^H DOSY NMR spectroscopy confirmed the presence of a single species in both cases (*D* = 1.22 × 10^−10^ m^2^ s^−1^ for the Pd(II) system and *D* = 1.20 × 10^−10^ m^2^ s^−1^ in the Pt(II) system) (Figures S17 and S21). HR ESI‐MS also confirmed the presence of the anticipated **[Pd(AD‐Bz)_2_](BF_4_)_2_** species in the first precipitate in a variety of charge states; *m/z* = 349.0779 **[Pd(AD‐Bz)_2_]^2+^** (calc. for C_32_H_32_N_8_O_4_Pd^2+^, 349.0801), *m/z* = 717.1586 [**Pd(AD‐Bz)_2_** + F^−^]^+^ (calc. for C_32_H_32_N_8_O_4_PdF^+^, 717.1552), *m/z* = 785.1590 [**Pd(AD‐Bz)_2_** + BF_4_^−^]^+^ (calc. for C_32_H_32_N_8_O_4_PdBF_4_^+^, 785.1498) (Figure S18). While [**Pt(AD‐Bz)_2_](BF_4_)_2_** was identified in the second precipitate, as expected, again in a variety of charge states; *m/z* = 393.6044 [**Pt(AD‐Bz)_2_**]^2+^ (calc. for C_32_H_32_N_8_O_4_Pt^2+^, 393.6078), *m/z* = 806.2119 [**Pt(AD‐Bz)_2_** + F^−^]^+^ (calc. for C_32_H_32_N_8_O_4_PtF^+^, 806.2219), *m/z* = 874.2180 [**Pt(AD‐Bz)_2_** + BF_4_^−^]^+^ (calc. for C_32_H_32_N_8_O_4_PtBF_4_^+^, 874.2222) (Figure S22). Further, crystals [51] of each complex were grown through diffusion of diethyl ether into a CH_3_CN/DMSO solution of the complex with single‐crystal X‐ray diffraction confirming the formation of the proposed species in a HT orientation, as expected [52, 53, 54] (SCXRD; *P* 2_1_/*n* for both complexes, Figure 1b,c). Differences in bond lengths between coordinating nitrogen atoms and each metal center were near‐zero, (N_triazole_─Pd bond length is 1.993 Å and the N_triazole_─Pt bond length is 1.995 Å; N_pyridine_─Pd bond length is 2.049 Å and the N_pyridine_─Pt bond length is 2.039 Å), meaning the dimensions of the complexes are effectively identical with any differences in behavior resulting solely from the identity of the metal ion itself.

With complexes in hand, suitable guests to assess both π and metallophilic interactions were designed and synthesized. For the sake of simplifying possible binding modes, two units of a recognizable motif were linked by an alkyl chain such that association should occur in a 1:1 host‐guest (HG) fashion. π interactions can be assessed using **G_2py_** (Figure 1e), which features pyrene units tethered by a triethylene glycol chain, and the naphthalene analogue, **G_2nap_** (Figure 1e), which had been previously synthesized within the group [55]. **G_2Pt_** (Figure 1e) was designed to probe metal–metal interactions. With the exception of the linker length, **G_2Pt_** is identical to a compound capable of metallophilic recognition by a dipalladium(II) metallocycle, as previously reported by Preston and coworkers [20]. Here, the hexane linker has been replaced with octane, in keeping with the lengths of **G_2py_** and **G_2nap_**. Initial modelling with Spartan24 [56], suggested this would be a suitable length to wrap around the complex such that each end of the guest can interact with either side of the host complex (Section S3). ^1^H DOSY diffusion coefficients of all HG complexes were in line with diffusion coefficients of the original hosts and guests, supporting their formation as intended and precluding the possibility of forming large amounts of polymeric species (Tables S1 and S2). We note that although these complexes don't bear traditional cavities or clefts for guest binding, we refer to them as “hosts” for simplicity.

### 2.2 Interactions With Organic Guests

*Choosing a solvent for ^1^H NMR spectroscopy*: ^1^H NMR spectroscopy was identified as a suitable technique to extract association constants and compare systems. The association constants needed to be large enough to extract meaningful differences between species but ideally less than 100,000 M^−1^ [57]. For all titrations, the host and guest were added as stock solutions in the solvent used in the respective titration. In general, guest was initially added from a 40 mM stock and beyond the 10th addition, an 80 mM stock was used to avoid large increases in the volume of the solution. A stock solution of host at twice the concentration of the original host solution was added throughout the titration to ensure the host concentration remained constant. A ^1^H NMR titration was first conducted adding **G_2py_** into a 4.5 mM solution of **[Pd(AD‐Bz)_2_](BF_4_)_2_** in CD_3_CN. Upfield shifts of most aromatic environments of **[Pd(AD‐Bz)_2_](BF_4_)_2_** were observed immediately, mostly stabilizing once only 1 equivalent of **G_2py_** was present (shown for H_e_ in blue in Figure 2). Due to the poor solubility of **G_2py_** in CD_3_CN, equivalences were calculated by NMR integration and precipitation of **G_2py_** unfortunately precluded the addition of the guest beyond 2.4 eq.

**FIGURE 2:**
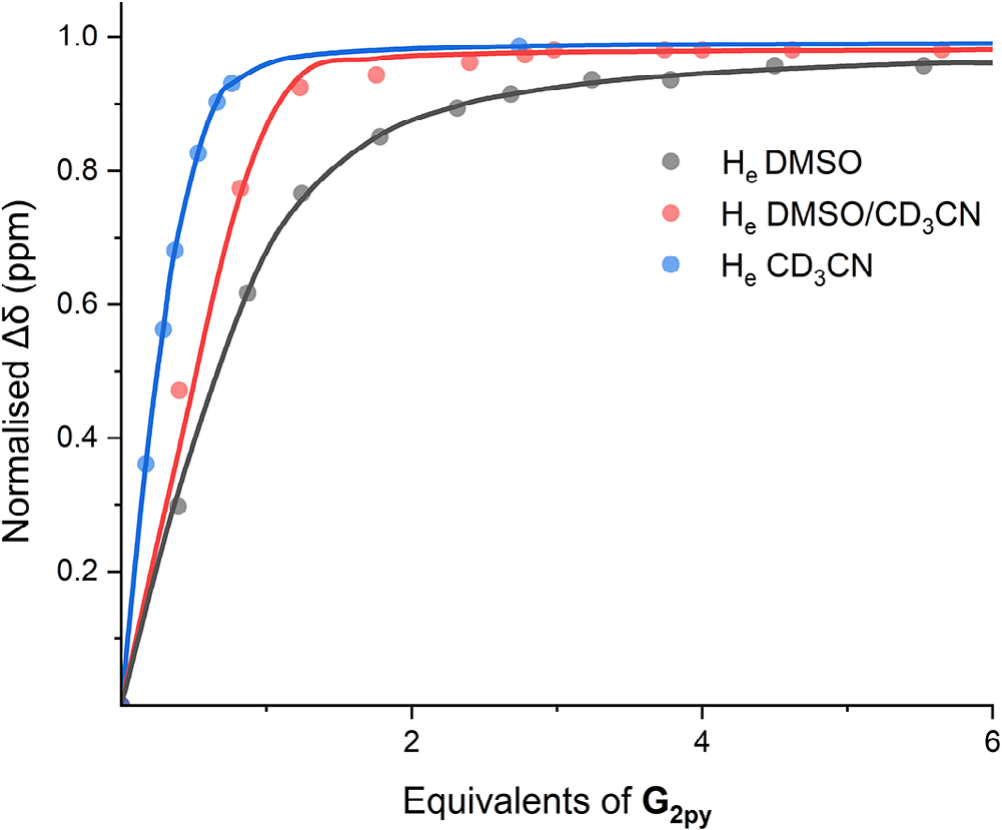
Demonstrates the normalized change in chemical shift of the H_e_ proton environment of **[Pd(AD‐Bz)_2_](BF_4_)_2_** as increasing equivalents of **G_2py_** were added across a variety of solvents: [D_6_]DMSO (grey), 1:1 CD_3_CN/[D_6_]DMSO (red) and CD_3_CN (blue). Data points depict the raw data and trendlines depict the fit provided by the Bindfit program [59].

Given the host (H), guest (G), and HG complexes are in fast exchange, the point where the change in chemical shifts of the “host” plateaus, represents predominance of the HG complex and is referred to as the “saturation point” herein. Although there are well‐established issues with the use of Job Plots and the mole‐ratio method in determining HG stoichiometry in many cases [58], 1:1 stoichiometry is clearly identifiable in this simple system given the association constant is significantly large. However, attempting to fit the obtained data to a 1:1 binding isotherm with Bindfit [59] produced a *K*_a_ estimated to be greater than 10^4^ M^−1^ and significant error, likely accentuated by issues with the poor solubility of **G_2py_**. The titration was repeated at a much lower concentration using a 0.05 mM solution of **[Pd(AD‐Bz)_2_](BF_4_)_2_**, however, useful data could not be extracted from such a dilute solution via ^1^H NMR spectroscopy with the instruments available to us.

Instead, it was proposed that based on our prior observations, changing the solvent to [D_6_]DMSO, for example, could assist in reducing the *K*_a_ values whilst improving solubility. In a 1:1 mixture of CD_3_CN and [D_6_]DMSO, using a more moderately concentrated, 1 mM solution of **[Pd(AD‐Bz)_2_](BF_4_)_2_**, the addition of **G_2py_** still resulted in immediately observable upfield shifts of the complex (demonstrated by H_e_ in red in Figure 2). A saturation point was delayed relative to the data observed using CD_3_CN, allowing for an estimation of *K*_a_ as 18,000 M^−1^, which is still fairly strong. When repeated in [D_6_]DMSO, the saturation point was delayed further (as illustrated by H_e_ in grey in Figure 2), to give data which provided a *K*_a_ value of 7,000 M^−1^ and reduced error (Table S13). Error associated with titration data presented herein is thoroughly detailed in the Supporting Information using different methods and predictive tools (Tables S13–S16). These results illustrate how solvent effects can significantly amplify or suppress the strength of π interactions in such systems.

*Association of* ***G_2py_***: To determine association constants between the host complex and **G_2py_**, [D_6_]DMSO was deemed the most suitable solvent. ^1^H NMR titrations were conducted in duplicate using 1 mM solutions of either metal complex, **[Pd(AD‐Bz)_2_](BF_4_)_2_** and **[Pt(AD‐Bz)_2_](BF_4_)_2_**, in [D_6_]DMSO. As previously mentioned, modeling the data obtained from the addition of **G_2py_** to **[Pd(AD‐Bz)_2_](BF_4_)_2_** with a 1:1 binding isotherm in Bindfit [59] produced a *K*_a_ of 7,000 M^−1^ (Table S13). In comparison, the respective data obtained from the platinum(II) analogue, **[Pt(AD‐Bz)_2_](BF_4_)_2_**, produced a significantly larger *K*_a_ value of 16,000 M^−1^ (Table S13). The observed difference in *K*_a_ values is much greater than the error predicted by Bindfit (Table S13) [59]. Further, modelling the data with other binding isotherm models and programs, namely Musketeer [60] and Bindtools [51], produces results in agreement with one another, and likewise suggests a 1:1 binding isotherm is suitable with minimal error (Table S13). Ultimately, we can be confident that the platinum(II) based system can form stronger π interactions than that of its palladium(II) counterpart. This trend can be observed visually in Figure 3, where proton environments associated with **[Pt(AD‐Bz)_2_](BF_4_)_2_** appear to reach a “saturation point” more quickly than the palladium(II) system.

**FIGURE 3:**
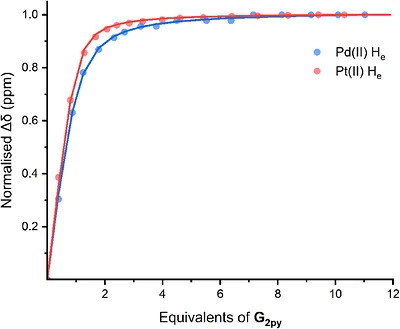
Compares the normalized change in chemical shift of the H_e_ proton environment of **[Pd(AD‐Bz)_2_](BF_4_)_2_** (blue) and **[Pt(AD‐Bz)_2_](BF_4_)_2_** (red) as increasing equivalents of **G_2py_** in [D_6_]DMSO. Data points depict the raw data and trendlines depict the fit provided by the Bindfit program [59].

From the independent titration data for each guest, it has been established that the change in chemical shift between the free host (δH) and HG complex (δHG) is comparable between the palladium(II) and platinum(II) systems (Table S18). Therefore, changes in chemical shifts of equivalent environments of **[Pd(AD‐Bz)_2_](BF_4_)_2_** and **[Pt(AD‐Bz)_2_](BF_4_)_2_** when guests are titrated into mixtures of the two systems should allow for an approximate qualitative comparison of their respective HG chemistry. HG mole fractions can also be calculated relative to their respective δH and δHG (extrapolated from Bindfit [59] modeling) for a more accurate comparison.

Increasing equivalents of **G_2py_** were added to a single solution of **[Pd(AD‐Bz)_2_](BF_4_)_2_** and **[Pt(AD‐Bz)_2_](BF_4_)_2_** in CD_3_CN, where association is known to be at its strongest (initially each at 3 mM). The H_a_ proton environment of the platinum(II) complex is observed via ^1^H NMR to move upfield more significantly than the corresponding H_a_ proton environment of the palladium(II) complex (represented by the red and blue lines of Figure 4a–f). Note that although H_a_ appears to overlap with other signals in Figure 4, the peaks can still be differentiated on the raw spectra (Figure S78). The HG mole fractions at each point were calculated based on these data and it was confirmed that the HG mole fraction of the platinum(II) complex is consistently larger than that of the palladium(II) complex (Figure 4g). The difference was most pronounced until 1 eq. of **G_2py_** had been added (Figure 4a–d), which was previously observed as the effective saturation point in CD_3_CN (Figure 2). Upon addition of 2 eq. of **G_2py_**, the HG mole fractions for both platinum(II) and palladium(II) complexes are close to 1; 0.97 and 0.98, respectively, as they have both effectively been saturated (Figure 4g).

**FIGURE 4:**
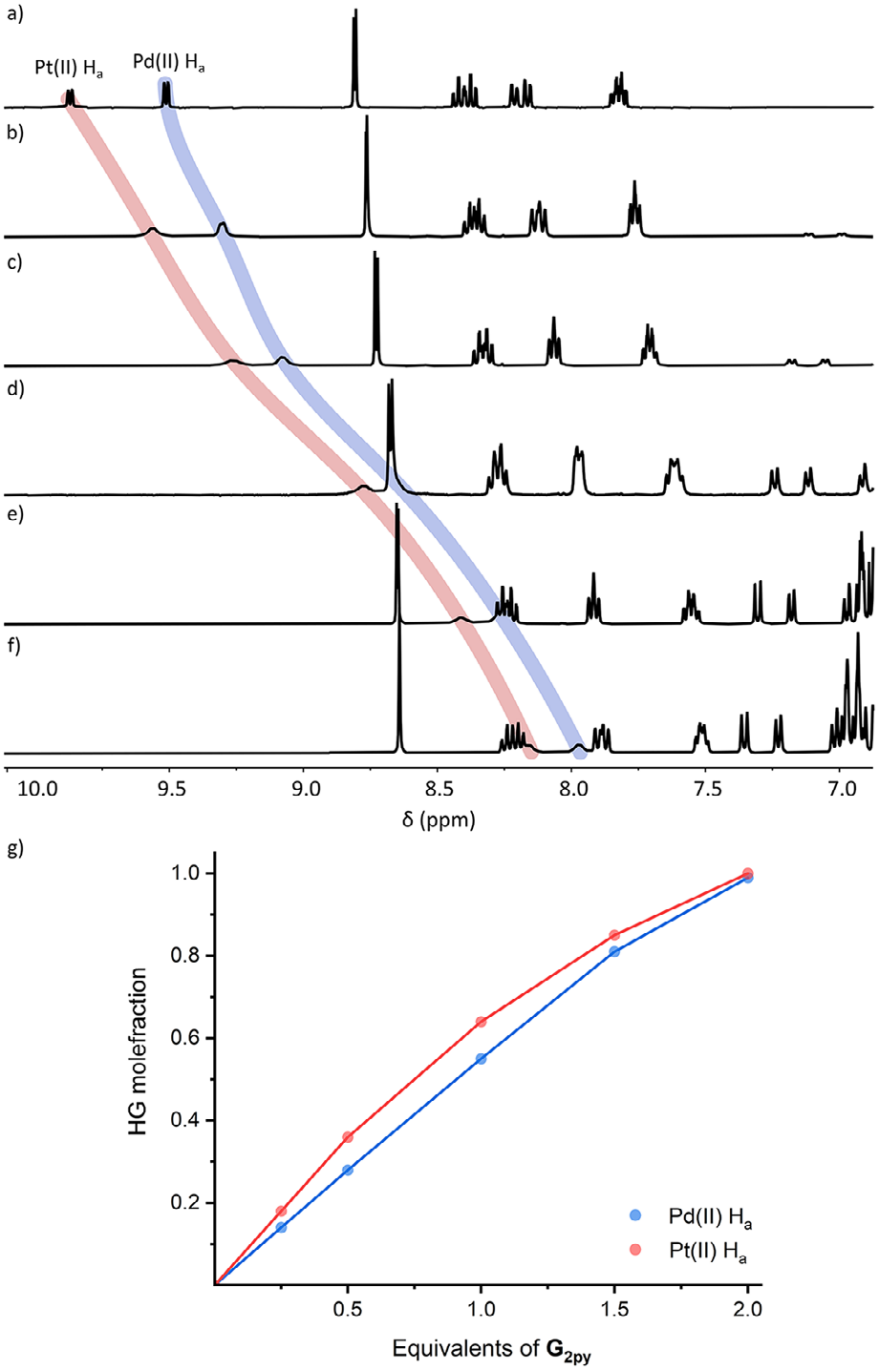
Stacked partial ^1^H NMR spectra (400 MHz, CD_3_CN, 298 K) highlighting the shifts of H_a_ in **[Pd(AD‐Bz)_2_](BF_4_)_2_** (blue) and **[Pt(AD‐Bz)_2_](BF_4_)_2_** (red) as increasing equivalents of **G_2py_** were added: (a) 0.00 eq., (b) 0.25 eq., (c) 0.50 eq., (d) 1.00 eq., (e) 1.50 eq., (f) 2.00 eq. of **G_2py_**, and (g) the change in HG mole fraction of **[Pd(AD‐Bz)_2_](BF_4_)_2_** (blue) and **[Pt(AD‐Bz)_2_](BF_4_)_2_** (red) as increasing equivalents of **G_2py_** were added in CD_3_CN. HG mole fractions have been calculated based on the chemical shift of H_a_ relative to the HG chemical shift predicted by the Bindfit program [59]. For a “zoomed” view of how we identified peak position where they partially coincide with other resonances, see Figure S78.

Comparable behavior was observed in [D_6_]DMSO, although less obvious as 2 eq. of **G_2py_** is not sufficient in saturating each complex in [D_6_]DMSO given the reduced association constants in this solvent (Figures S75 and S76). In competition, although not particularly selective, the platinum(II) complex can therefore preferentially bind **G_2py_** more strongly than the palladium(II) complex. While these data from the competition experiments rely in part on extrapolated δHG values and must therefore be viewed with caution, we note that they align well with the calculated binding affinities, and the overall visible differences between the Pt(II) and Pd(II) environments as the competition experiment progresses. Namely, the gradient associated with the Pt(II) HG mole fraction is steeper, indicating a more rapid approach to saturation (Figure 4g).

*Association of* ***G_2nap_*** *and* ***G_py_***: As can be expected from the smaller π surface of naphthalene compared to pyrene, association constants with **G_2nap_** were much lower than with **G_2py_**. ^1^H NMR titration experiments (performed in duplicate) between **G_2nap_** and the two hosts, respectively, confirmed this, with 1 mM solutions of metal complexes in [D_6_]DMSO providing *K*_a_ values of 30 M^−1^ for both the palladium(II) and platinum(II) complexes (calculated using Bindfit20 [59]) (Table S15).

Repeating the titrations in CD_3_CN, produced *K*_a_ values that were stronger (*K*_a_ = 500 M^−1^), but still identical to one another irrespective of which metal complex was present (Table S14). The association constants are simply too low to read into this behavior, but it is worth noting that, not unexpectedly, the difference between metal ions may not be as significant in more weakly associating systems. Similarly, having synthesized **G_py_** (Figure 1e) as a variant of **G_2py_** with a single pyrene motif, we additionally carried out similar experiments with **G_py_** in CD_3_CN. The binding here was again too weak to observe a trend, with little difference between the complexes and relatively high error, likely due to the poor solubility of **G_py_** in CD_3_CN (*K*_a_ = 1261 M^−1^ for **[Pd(AD‐Bz)_2_](BF_4_)_2_** and *K*_a_ = 917 M^−1^ for **[Pt(AD‐Bz)_2_](BF_4_)_2_**) (Tables S11 and S12). This supports the design of the original guests, which allow for a more accurate comparison between systems.

*Computational analysis*: Homodesmotic analysis of HG complexes optimized via density functional theory (DFT) [61] calculations produced varied results between increasing levels of PBE‐based theory (Figures S88–S90) [62, 63, 64]. Resnati, Frontera, and coworkers [44] previously established that consideration of dispersion forces was necessary for π‐stacking systems like ours. With PBE0‐D4/def2‐TZVP theory, which does consider these dispersion forces, the formation of the Pt(II)‐based HG complexes with **G_2py_** and **G_2nap_** was calculated to be energetically comparable to the Pd(II)‐based counterparts, especially when compared to the pronounced effect we observe experimentally (Tables S22–S25). Further, a higher dispersion contribution is calculated for **G_2nap_** compared to **G_2py_**, which is also inconsistent with our experimental results (Tables S22–S25). The only energetic differences observed via molecular dynamics simulations (GFN2‐*xTB* MD) [65] of HG complexes with **G_2py_** and **G_2nap_** were associated with insignificant sliding of the guest and again showed few differences in the behavior of Pd(II) and Pt(II) complexes (Section S3.5). Interestingly, performing the same DFT analysis on the Pt(II) and Pd(II) HG system with pyrene, rather than **G_2py_**, produced more consistent results in which the Pt(II) HG complex was approximately 8 kJ/mol more favorable than the Pd(II) analogue (Table S27). This is in line with the prior work of Resnati, Frontera, and coworkers [44], indicating that the calculations (at this level of theory) only become less reliable when greater complexity is incorporated into the HG system. This is something to keep in mind when using computational analysis to assess the potential stability of targets, which may depend on differentiation of such interactions with planar systems.

Despite several attempts, crystals of either complex with **G_2py_** could not be obtained, likely because of the extremely favorable crystal packing the host complex can establish with itself, aided by the methoxybenzene sidearms. A very similar complex, **[Pd(AD‐PEG)_2_](BF_4_)_2_** [55], had been previously synthesized within the Preston group, with the only difference being substitution of the methoxybenzene side arms with a monoethylene glycol solubilizing chain. We had also synthesized **G_py_** (Figure 1e) as a variant of **G_2py_** with only a single pyrene motif and a monoethylene glycol solubilizing chain, which we suspected may crystallize more easily. Diffusion of diethyl ether into a CH_3_CN/DMSO solution of **G_py_** and **[Pd(AD‐PEG)_2_](BF_4_)_2_** finally resulted in crystals of a comparable HG complex.

The solution of the single‐crystal X‐ray diffraction depicts slightly offset stacking between the pyrene molecules with the center of the palladium(II) complex, where the interplanar distance of the two is 3.340 Å, in line with literature precedent [55] for similar π interactions (SCXRD; Pbcn, Figure 5a). Interestingly, the crystal packing reveals metal–metal interactions between Pd(II) centers of neighboring [Pd(AD‐PEG)_2_]^2+^ units, where **G_py_** is interspersed only between every second unit of the complex (Figure 5b).

**FIGURE 5:**
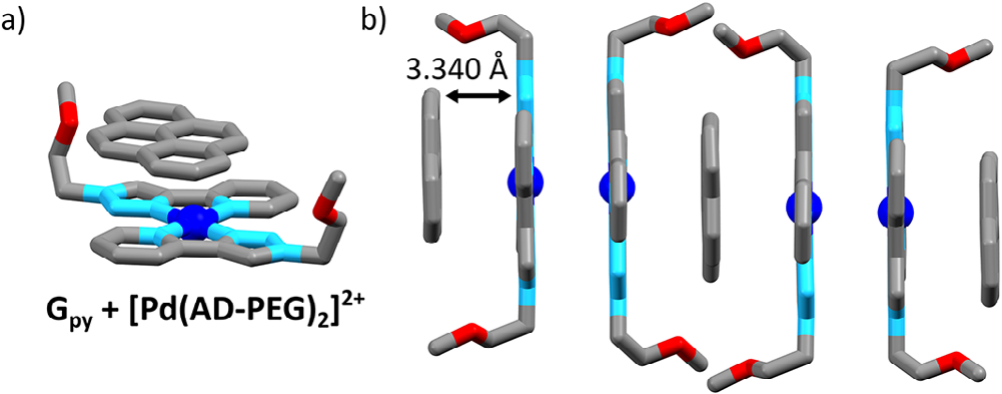
(a) X‐ray crystal structure of **G_py_** and **[Pd(AD‐PEG)_2_]^2+^**, and (b) a selected expanded view of the crystal packing of this system. The solubilizing chain of **G_py_** was significantly disordered, so it has been omitted for clarity.

### 2.3 Interactions With a Diplatinate Guest

In addition to π interactions, this study probed the potential differences between Pd(II) and Pt(II) centers in establishing interactions with the diplatinate guest, **G_2Pt_**. ^1^H NMR titrations were conducted in duplicate using 1 mM solutions of either metal complex, **[Pd(AD‐Bz)_2_](BF_4_)_2_** and **[Pt(AD‐Bz)_2_](BF_4_)_2_**, in [D_6_]DMSO with the addition of **G_2Pt_**. Fitting the data obtained from the **[Pd(AD‐Bz)_2_](BF_4_)_2_** with a 1:1 binding isotherm in Bindfit [59] produced a *K*_a_ of 50 M^−1^ (Table S16). In comparison, the analogous data obtained with **[Pt(AD‐Bz)_2_](BF_4_)_2_**, produced a larger *K*_a_ value of 100 M^−1^ (Table S16). As with titrations of the **G_2py_** guest, the values were consistent across multiple programs and were associated with minimal error. Unfortunately, titrations in CD_3_CN, which should give larger *K*_a_ values, could not be performed due to poor solubility of **G_2Pt_**. Despite these interactions being relatively weak, we observe a significant difference between systems in line with our findings with **G_2py_** and previous work by Crowley and Bosnich [37].

A competition experiment was performed in which increasing equivalents of **G_2Pt_** was added to a solution of **[Pd(AD‐Bz)_2_](BF_4_)_2_** and **[Pt(AD‐Bz)_2_](BF_4_)_2_** in [D_6_]DMSO. As previously, H_a_ of the palladium(II) complex appears to overlap with other signals in Figure 6, however the peaks can still be easily differentiated on the raw spectra. Although not immediately obvious from the ^1^H NMR spectra (Figure 6a–f) given the weak nature of the **G_2Pt_** association, the HG mole fraction of the platinum(II) complex is consistently higher than that of the palladium(II) complex (Figure 6g). When in competition with one another, the platinum(II) complex can bind **G_2Pt_** more strongly. This is in line with what the ^1^H NMR titration data originally suggested; the platinum(II) complex can interact with a diplatinate guest more strongly than its palladium(II) counterpart.

**FIGURE 6:**
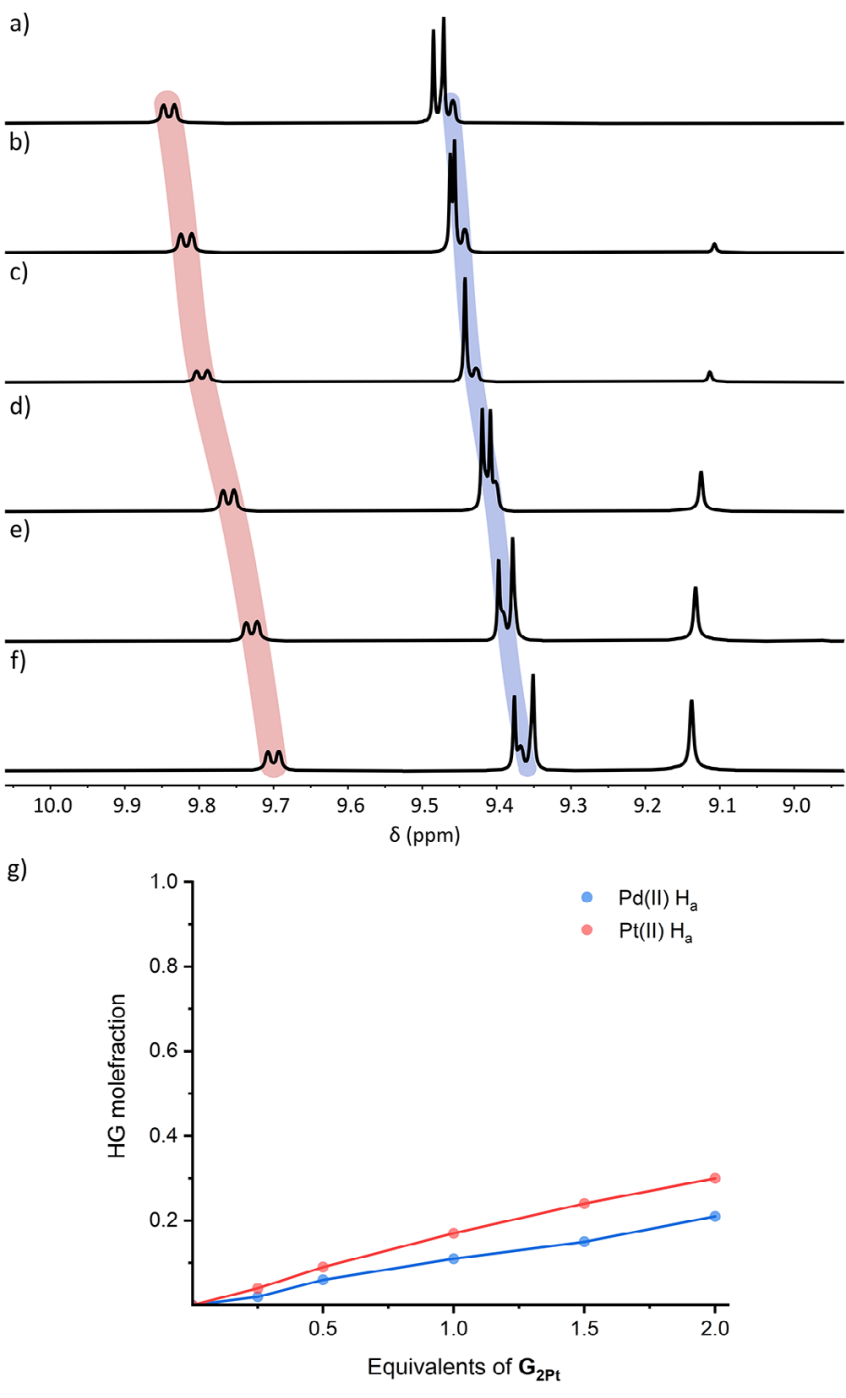
Stacked partial ^1^H NMR spectra (400 MHz, [D_6_]DMSO, 298 K) highlighting the shifts of H_a_ in **[Pd(AD‐Bz)_2_](BF_4_)_2_** (blue) and **[Pt(AD‐Bz)_2_](BF_4_)_2_** (red) as increasing equivalents of **G_2Pt_** were added: (a) 0.00 eq., (b) 0.25 eq., (c) 0.50 eq., (d) 1.00 eq., (e) 1.50 eq., and (f) 2.00 eq. of **G_2Pt_** compared with (g) the change in HG mole fraction of **[Pd(AD‐Bz)_2_](BF_4_)_2_** (blue) and **[Pt(AD‐Bz)_2_](BF_4_)_2_** (red) as increasing equivalents of **G_2Pt_** were added in [D_6_]DMSO. HG mole fractions have been calculated based on the chemical shift of H_a_ relative to the HG chemical shift predicted by the Bindfit program [59].

Computational analysis again produced varied results across different levels of theory, with all observed energy differences of HG complexes being extremely small (Tables S23–S26). Generally, metal–metal interactions would more obviously manifest crystallographically. Similar issues were encountered in obtaining crystals of the target complexes. Following literature procedures [19, 66], we synthesized **G_Pt_** (Figure 1e), a variant of **G_2Pt_**, which features only a single platinum(II) center and no tether. A platinum variant of the **[Pd(AD‐PEG)_2_](BF_4_)_2_** [55] complex was also synthesized (Figures S23–S25). Diffusion of diethyl ether into separate CH_3_CN/DMSO solutions of **[Pd(AD‐PEG)_2_](BF_4_)_2_** and **[Pt(AD‐PEG)_2_](BF_4_)_2_** each with **G_Pt_**, finally resulted in crystals of representative HG complexes.

The solutions for each SCXRD (*P*2_1_, Figure 7a,c) are extremely similar, depicting surprisingly offset stacking between **G_Pt_** and each host complex, with an interplanar distance of 3.372 and 3.376 Å respectively, but a Pd(II)‐Pt(II) distance of 3.660 Å (82% of the sum of the van der Waals radii [67]) and a Pt(II)‐Pt(II) distance of 3.650 Å (80% of the sum of the van der Waals radii [67]). The interplanar distance is in line with literature precedent but as a consequence of the offset stacking, the metal–metal distances are slightly longer than the expected 3.3–3.4 Å [14, 18, 19, 35, 37]. It is possible that in our simple system with free planar metal faces and no true cavities for guest binding that the crystal packing may not be truly representative of what we observe in solution. Nonetheless, the metal–metal distances are still less than the sum of their van der Waals radii, and coupled with the solution‐phase data, there is clearly some interaction between the platinate guest and the host complexes. However, if metal–metal interactions do truly exist they are certainly weak, so irrespective of the metal center, it is unlikely they would play a significant role in directing self‐assemblies.

**FIGURE 7:**
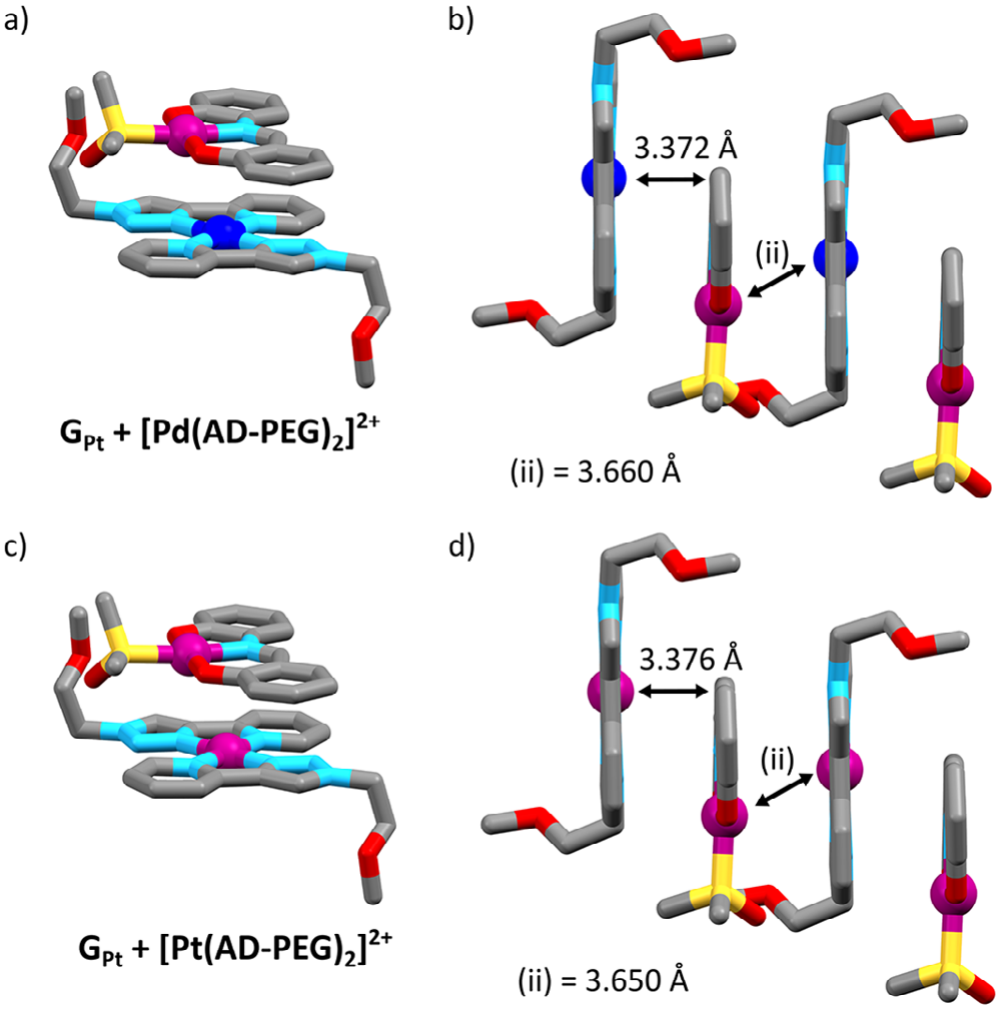
(a) X‐ray crystal structure of **G_Pt_** and **[Pd(AD‐PEG)_2_]^2+^** and (c) **G_Pt_** and **[Pt(AD‐PEG)_2_]^2+^** compared to (b) and (d) a selected expanded view of the crystal packing of each system.

## 3 Conclusion

The palladium(II) and platinum(II) model complexes, **[Pd(AD‐Bz)_2_](BF_4_)_2_** and **[Pt(AD‐Bz)_2_](BF_4_)_2_**, were used in this study to probe potential differences in their interactions with organic aromatic guests and platinate guests. Provided binding is strong, ^1^H NMR titrations and competition experiments revealed platinum(II)‐containing hosts can form interactions with organic guests, **G_2py_**, roughly twice the strength of palladium(II) analogues. π interactions between a pyrene motif and a palladium(II) planar surface were additionally observed crystallographically in a representative HG complex. Computational analysis was varied but ultimately could not predict the observed differences between the Pd(II) and Pt(II) HG complexes. This is important to recognize in the future when using similar computational techniques to predict or post‐rationalize the behavior of similar systems.

Metal–metal interactions were also investigated using a diplatinate guest, **G_2Pt_**. These interactions were again notably more significant for platinum(II) hosts than their palladium(II) analogues. As before, computational analysis could not predict the observed behavior. Irrespective of the host, the metal‐metal interactions are consistently weak, and unlikely to play a significant role in affecting the HG chemistry or conformation of similar systems.

Combined, we have established that the identity of the metal ion in planar species can indeed affect their relative strength of interactions with organic and inorganic guests. We suggest that this difference could simply arise from the greater electron density of platinum(II) relative to palladium(II). We hope that in the future, this knowledge can either inform the design or account for the behavior of metallo‐supramolecular systems which similarly rely on interactions between planar species. For example, if faced with a relatively simple equilibrium mixture of palladium(II)‐based products in which the target features strong π interactions, perhaps the alternate use of platinum(II) could sufficiently favor equilibria toward the targeted product, eliminating the formation of side‐products.

## Conflicts of Interest

The authors declare no conflicts of interest

## Supporting information




**Supporting File 1**: asia71018‐sup‐0001‐SuppMat.xyz
The authors have cited additional references within the Supporting information [20, 49, 50, 51, 55–87].

**Supporting File 2**: asia71018‐sup‐0002‐SuppMat.docx

**Supporting File 3**: asia71018‐sup‐0003‐SuppMat.cif

## Data Availability

The data that supports the findings of this study are available in the Supporting Information of this article.
