# Supplementary material for: Relative Influence of Pd(II) and Pt(II) Metal Centers on the Strength of Interactions Between Planar Species
Source: Chem Asian J. 2026 Oct 18;21(20):e71018. doi: 10.1002/asia.71018 (PMC13677020; doi:10.1002/asia.71018)
Supplement: Supplementary file 1 [file ASIA-21-e71018-s002.docx]

Supporting Information

*Jess L. Algar,^a,*^ Rosemary J. Goodwin,^a^ Dan Preston^a,*^*

*^a^Research School of Chemistry, The Australian National University, Canberra, ACT 2600, Australia.*

***jessica.algar@anu.edu.au**

***daniel.preston@anu.edu.au**

**Contents**

[1.	Experimental	5](#_Toc239945070)

[1.1.	General	5](#_Toc239945071)

[1.1.1.	NMR pulse programs and parameters	5](#_Toc239945072)

[1.2.	Ligands	6](#_Toc239945073)

[1.2.1.	AD-Bz	6](#_Toc239945074)

[1.3.	Guests	7](#_Toc239945075)

[1.3.1.	G_2nap_	7](#_Toc239945076)

[1.3.2.	G_2py_	8](#_Toc239945077)

[1.3.3.	G_2Pt_	10](#_Toc239945078)

[1.3.4.	G_py_	11](#_Toc239945079)

[1.4.	Complexes	13](#_Toc239945080)

[1.4.1.	[Pd(AD-Bz)_2_](BF_4_)_2_	13](#_Toc239945081)

[1.4.2.	[Pt(AD-Bz)_2_](BF_4_)_2_	15](#_Toc239945082)

[1.4.3.	[Pt(AD-PEG)_2_](BF_4_)_2_	17](#_Toc239945083)

[2.	Host-Guest Chemistry	19](#_Toc239945084)

[2.1.	Mass spectrometry	19](#_Toc239945085)

[2.2.	NMR data	24](#_Toc239945086)

[2.2.1.	[G_2py_ ⊂ Pd(AD-Bz)_2_](BF_4_)_2_	24](#_Toc239945087)

[2.2.2.	[G_2py_ ⊂ Pt(AD-Bz)_2_](BF_4_)_2_	25](#_Toc239945088)

[2.2.3.	[G_2nap_ ⊂ Pd(AD-Bz)_2_](BF_4_)_2_	26](#_Toc239945089)

[2.2.4.	[G_2nap_ ⊂ Pt(AD-Bz)_2_](BF_4_)_2_	26](#_Toc239945090)

[2.2.5.	[G_2Pt_ ⊂ Pd(AD-Bz)_2_](BF_4_)_2_	27](#_Toc239945091)

[2.2.6.	[G_2Pt_ ⊂ Pt(AD-Bz)_2_](BF_4_)_2_	28](#_Toc239945092)

[2.3.	^1^H DOSY NMR Summary	28](#_Toc239945093)

[2.4.	NMR titration data	29](#_Toc239945094)

[2.4.1.	General titration information	29](#_Toc239945095)

[2.4.2.	[G_2py_ ⊂ Pd(AD-Bz)_2_](BF_4_)_2_	30](#_Toc239945096)

[2.4.3.	[G_2py_ ⊂ Pt(AD-Bz)_2_](BF_4_)_2_	32](#_Toc239945097)

[2.4.4.	[G_2nap_ ⊂ Pd(AD-Bz)_2_](BF_4_)_2_	35](#_Toc239945098)

[2.4.5.	[G_2nap_ ⊂ Pt(AD-Bz)_2_](BF_4_)_2_	38](#_Toc239945099)

[2.4.6.	[G_2Pt_ ⊂ Pd(AD-Bz)_2_](BF_4_)_2_	41](#_Toc239945100)

[2.4.7.	[G_2Pt_ ⊂ Pt(AD-Bz)_2_](BF_4_)_2_	43](#_Toc239945101)

[2.4.8.	[G_py_ ⊂ Pd(AD-Bz)_2_](BF_4_)_2_	46](#_Toc239945102)

[2.4.9.	[G_py_ ⊂ Pt(AD-Bz)_2_](BF_4_)_2_	48](#_Toc239945103)

[2.5.	Calculation of Binding Constants (1:1 Model)	50](#_Toc239945104)

[2.5.1.	General Information	50](#_Toc239945105)

[2.5.2.	G_2py_	50](#_Toc239945106)

[2.5.3.	G_2nap_	50](#_Toc239945107)

[2.5.4.	G_2Pt_	50](#_Toc239945108)

[2.6.	Competition Experiments	51](#_Toc239945109)

[2.6.1.	General Information	51](#_Toc239945110)

[2.6.2.	G_2py_	51](#_Toc239945111)

[2.6.3.	G_2nap_	54](#_Toc239945112)

[2.6.4.	G_2Pt_	57](#_Toc239945113)

[3.	Calculations	58](#_Toc239945114)

[3.1.	Structure optimisation	58](#_Toc239945115)

[3.2.	Electrostatic potential maps	60](#_Toc239945116)

[3.3.	Analysis of structures	60](#_Toc239945117)

[3.3.1.	Analysis of [G_2_ ⊂ M(AD-Bz*_anti_*)_2_]^2+^ complexes	61](#_Toc239945118)

[3.3.2.	Analysis of [G_2_ ⊂ M(AD-Bz*_syn_*)_2_]^2+^ complexes	62](#_Toc239945119)

[3.3.3.	Analysis of [pyrene ⊂ M(AD-model)_2_]^2+^ complexes	62](#_Toc239945120)

[3.4.	Homodesmotic analysis	62](#_Toc239945121)

[3.4.1.	Analysis of [G_2py_ ⊂ M(AD-Bz*_anti_*)_2_]^2+^ homodesmotic assemblies	62](#_Toc239945122)

[3.4.2.	Analysis of [G_2py_ ⊂ M(AD-Bz*_syn_*)_2_]^2+^ homodesmotic assemblies	63](#_Toc239945123)

[3.4.3.	Analysis of [G_2nap_ ⊂ M(AD-Bz*_anti_*)_2_]^2+^ homodesmotic assemblies	64](#_Toc239945124)

[3.4.4.	Analysis of [G_2nap_ ⊂ M(AD-Bz*_syn_*)_2_]^2+^ homodesmotic assemblies	64](#_Toc239945125)

[3.4.5.	Analysis of [G_2Pt_ ⊂ M(AD-Bz*_anti_*)_2_]^2+^ homodesmotic assemblies	65](#_Toc239945126)

[3.4.6.	Analysis of [pyrene ⊂ M(AD-model)_2_]^2+^ homodesmotic assemblies	65](#_Toc239945127)

[3.5.	Molecular dynamics simulations	66](#_Toc239945128)

[3.5.1.	Dynamic equilibrium of [G_2py_ ⊂ Pd(AD-Bz)_2_]^2+^	66](#_Toc239945129)

[3.5.2.	Dynamic equilibrium of [G_2py_ ⊂ Pt(AD-Bz)_2_]^2+^	68](#_Toc239945130)

[3.5.3.	Dynamic equilibrium of [G_2nap_ ⊂ Pd(AD-Bz)_2_]^2+^	69](#_Toc239945131)

[3.5.4.	Dynamic equilibrium of [G_2nap_ ⊂ Pt(AD-Bz)_2_]^2+^	70](#_Toc239945132)

[4.	Crystallography	71](#_Toc239945133)

[4.1.	Experimental data	71](#_Toc239945134)

[5.	References	77](#_Toc239945135)

# 1. Experimental

## 1.1. General

Unless otherwise stated, all reagents were purchased from commercial sources and used without further purification, except for:

(ethane-1,2-diylbis(oxy))bis(ethane-2,1-diyl) bis(4-methylbenzenesulfonate)^[1]^

triethylene glycol ditosylate^[1]^

**AD-PEG^[2]^**

[((*E*-2-((2-oxidobenzylidene)amino)-phenolate)(DMSO)platinum(II)] (**G_Pt_**)^[3]^

which were synthesised according to literature procedures.

Solvents were laboratory reagent grade. Petroleum ether refers to the fraction of petrol boiling in the range 40 – 60 °C. Abbreviations: dichloromethane (DCM), ethylenediaminetetraacetate (EDTA), tetrahydrofuran (THF), triethylamine (TEA), dimethyl sulfoxide (DMSO), dimethylformamide (DMF). ^1^H and ^13^C NMR spectra were recorded on either a Bruker Avance 400 MHz, 600 MHz or 700 MHz spectrometer. Chemical shifts are reported in parts per million and referenced to *residual solvent peaks*, labelled in spectral figures for the fully deuterated solvent used (CDCl_3_: ^1^H δ 7.26 ppm, ^13^C δ 77.16 ppm; [D_6_]DMSO: ^1^H δ 2.50 ppm; [D_3_]acetonitrile: ^1^H δ 1.94 ppm). Coupling constants (*J*) are reported in Hertz (Hz). Standard abbreviations indicating multiplicity were used as follows: m = multiplet, q = quartet, quin = quintet, t = triplet, dt = double triplet, d = doublet, dd = double doublet, s = singlet, br = broad. Electrospray mass spectra (HR ESI-MS) were collected on a Bruker micrOTOF-Q spectrometer or a Waters Synapt G2-S1 HDMS spectrometer.

**CAUTION: WHILE NO PROBLEMS WERE ENCOUNTERED DURING THIS WORK, AZIDES ARE EXPLOSIVE AND CARE SHOULD BE TAKEN WHEN DEALING WITH THEM.**

### 1.1.1. NMR pulse programs and parameters

All ^1^H 1D TOCSY NMR spectra were obtained using the selmlgp pulse program^[4]^ within Topspin.

All ^1^H 2D NOESY NMR spectra were obtained using the noesygpphpp pulse program^[5, 6]^ within Topspin. Relaxation time was set to 200 ms.

All ^1^H DOSY NMR spectra were obtained using the convection compensated dstebpgp3s pulse sequence^[7, 8]^ within Topspin. The spectra were obtained with δ = 2.0 ms, Δ = 100 ms, and g = 2% – 95% in [D_6_]DMSO. Processing was carried out in MestreNova.

## 1.2. Ligands

### 1.2.1. AD-Bz

The combination of 1-(bromomethyl)-3,5-dimethoxybenzene (0.500 g, 2.16 mmol) and NaN_3_ (0.141 g, 2.16 mmol) in a solution of H_2_O (10 mL) and acetone (40 mL) was stirred at room temperature for 18 hours. DCM (100 mL) was added to the solution and the organic layer washed with water (2 × 50 mL) and the solvent removed under vacuum. The residue was dissolved in a solution of DMF (7 mL) and H_2_O (1 mL). 2-Ethynylpyridine (0.256 g, 2.48 mmol), sodium ascorbate (0.205 g, 1.04 mmol) and Cu(SO_4_)_2_∙5H_2_O (0.259 g, 1.04 mmol) were added to the solution and the reaction was stirred at room temperature overnight under nitrogen. After adding DCM (100 mL) and 0.1 M aqueous EDTA/NH_4_OH (0.1 M, 100 mL) and stirring vigorously for 30 minutes, the organic layer was washed with water (5 × 100 mL), and solvent removed under vacuum. Purification by column chromatography on silica (DCM to 1:9 acetone/DCM) gave the product as a white crystalline solid (452 mg, 1.53 mmol, 74%). ^1^H NMR (400 MHz, CDCl_3_, 298 K) *δ*: 8.54 (1H, d, *J* = 4.6 Hz, H_a_), 8.18 (1H, d, *J* = 8.0 Hz, H_d_), 8.09 (1H, s, H_e_), 7.77 (1H, ddd, *J* = 8.5 Hz, 7.8 Hz, 1.8 Hz, H_c_), 7.21 (1H, ddd, *J* = 7.8 Hz, 4.6 Hz, 1.8 Hz, H_b_), 6.46 (2H, d, *J* = 2.2 Hz, H_g_), 6.42 (1H, t, *J* = 2.2 Hz, H_h_), 5.49 (2H, s, H_f_), 3.76 (6H, s, H_i_). ^13^C NMR (100 MHz, CDCl_3_, 298 K) *δ*: 161.5, 150.2, 149.3, 148.6, 137.2, 136.5, 123.0, 122.2, 120.4, 106.5, 100.8, 55.6, 54.6. HR ESI-MS (methanol) *m/z* = 297.1411 [**AD-Bz** + H^+^]^+^ (calc. for C_16_H_17_N_4_O_2_^+^, 297.1352), 319.1189 [**AD-Bz** + Na^+^]^+^ (calc. for C_16_H_16_N_4_O_2_Na^+^, 319.1171). IR *v* (cm^-1^) 3142, 3087, 3051, 3008, 2939, 2839, 1596, 1571, 1549, 1472, 1431, 1420.

Figure S1 ^1^H NMR spectrum (400 MHz, CDCl_3_, 298 K) of AD-Bz.

Figure S2 ^13^C NMR spectrum (100 MHz, CDCl_3_, 298 K) of AD-Bz.

## 1.3. Guests

### 1.3.1. G_2nap_

2-Naphthol (0.629 g, 4.36 mmol), triethylene glycol ditosylate^[1]^ (1.00 g, 2.18 mmol) and potassium carbonate (0.603 g, 4.36 mmol) was dissolved in DMF (20 mL) and the mixture was heated at 80 °C overnight. The reaction mixture was diluted with DCM (100 mL) and the organic layer was washed with water (3 × 100 mL). The solvents were removed under reduced pressure. The residue was purified by column chromatography (eluting with DCM) to give the desired product as a white crystalline powder after removal of solvent. Yield: 0.609 g (69%). ^1^H NMR (400 MHz, CDCl_3_, 298 K) δ: *integration given per ‘half’ of the guest* 7.76 (d, *J* = 8.1 Hz, 1H, H_3_), 7.73-7.69 (m, 2H, H_2_, H_6_), 7.42 (t, *J* = 7.5 Hz, 1H, H_5_), 7.33 (t, *J* = 7.5 Hz, 1H, H_4_), 7.18 (dd, *J* = 8.9 Hz, 2.5 Hz, 1H, H_1_), 7.13 (s, 1H, H_7_), 4.25 (t, *J* = 4.7 Hz, 2H, H_8_), 7.33 (t, *J* = 5.0 Hz, 2H, H_9_), 3.81 (s, 2H, H_10_); ^13^C NMR (100 MHz, CDCl_3_, 298 K) δ: 156.9, 134.6, 129.5, 129.2, 127.8, 126.9, 126.5, 123.8, 119.1, 106.9, 71.1, 70.0, 67.6; *D* (× 10^-10^ m^2^ s^-1^, 400 MHz, [D_6_]DMSO, 298 K) = 1.99; HR ESI-MS (CDCl_3_/MeOH) *m/z* = 425.1731 [**G_2nap_** + Na^+^]^+^ (calc. for C_26_H_26_O_4_Na, 425.1729); IR *v* (cm^‑1^) 3058, 2979, 2930, 2899, 2868, 1626, 1255, 1215, 1131, 1116, 836, 741.

Figure S3 ^1^H NMR spectrum (400 MHz, CDCl_3_, 298 K) of G_2nap_.

Figure S4 ^13^C NMR spectrum (100 MHz, CDCl_3_, 298 K) of G_2nap_.

Figure S5 ^1^H DOSY NMR spectrum (400 MHz, [D_6_]DMSO, 298 K) of G_2nap_.

### 1.3.2. G_2py_

Triethylene glycol ditosylate^[1]^ (200 mg, 0.436 mmol), pyrene-1-ol (286 mg, 1.31 mmol) and potassium carbonate (145 mg, 1.05 mmol) were combined in DMF (10 mL) and heated at 80 °C for 18 h. The mixture was diluted with DCM (20 mL) and the organic phase the organic layer was washed with water (5 × 100 mL), and solvent removed under vacuum. Purification by column chromatography on silica (DCM to 1:9 acetone/DCM) gave the product as a brown oil (101 mg, 0.182 mmol, 42%). ^1^H NMR (400 MHz, CDCl_3_, 298 K) *δ*: *integration given per ‘half’ of the guest* 8.49 (d, *J* = 9.1 Hz, 1H, H_2_), 8.11-8.03 (m, 2H, H_3_, H_5_), 8.02-7.85 (m, 5H, H_1_, H_4_, H_6_, H_7_, H_8_), 7.41 (d, *J* = 8.5 Hz, 1H, H_9_), 4.39 (t, *J* = 4.3 Hz, 2H, H_10_), 4.04 (t, *J* = 4.3 Hz, 2H, H_11_), 3.88 (s, 2H, H_12_); ^13^C NMR (100 MHz, CDCl_3_, 298 K) *δ*: 152.9, 131.74, 131.69, 127.3, 126.4, 126.1, 125.8, 125.48, 125.45, 125.1, 124.9, 124.3, 124.2, 121.4, 120.6, 109.5, 71.2, 70.1, 68.7; *D* (× 10^-10^ m^2^ s^-1^, 400 MHz, [D_6_]DMSO, 298 K) = 1.71; HR ESI-MS (methanol) *m/z* = 551.2231 [**G_2py_** + H^+^]^+^ (calc. for C_38_H_31_O_4_^+^, 551.2217); IR *v* (cm^-1^) 3041, 2922, 2871, 1627, 1598, 1507, 1488, 1453, 1433, 1407.

Figure S6 ^1^H NMR spectrum (400 MHz, CDCl_3_, 298 K) of G_2py_.

Figure S7 ^13^C NMR spectrum (100 MHz, CDCl_3_, 298 K) of G_2py_.

Figure S8 ^1^H DOSY NMR spectrum (400 MHz, [D_6_]DMSO, 298 K) of G_2py_.

### 1.3.3. G_2Pt_

An analogous preparation was used as in previous work by Preston and co-workers.^[9]^ [((*E*-2-((2-oxidobenzylidene)amino)-phenolate)(DMSO)platinum(II)]^[3]^ (252 mg, 0.520 mmol) was stirred in acetonitrile (6 mL) under a N_2_ atmosphere. A solution of 1,8-diaminooctane (30.0 mg, 0.208 mmol) in acetonitrile (1 mL) was added. The reaction was heated under N_2_ at reflux for 18 hours. After cooling to room temperature, the solid was collected via filtration, and washed with acetonitrile (4 × 1 mL) to give the product as an orange solid (133 mg, 0.139 mmol, 67%).

The guest is relatively insoluble in CDCl_3_, so ^1^H NMR data is provided in both CDCl_3_ and [D_6_]DMSO. ^1^H NMR (400 MHz, CDCl_3_, 298 K) *δ*: *integration given per ‘half’ of the guest* 8.28 (s, 1H, H_5_), 7.62 (d, *J* = 8.3 Hz, 1H, H_4_), 7.45-7.32 (m, 2H, H_6_, H_8_), 7.09-7.06 (m, 2H, H_2_, H_9_), 6.96 (d, 1H, *J* = 8.4 Hz, H_1_), 6.73-6.65 (m, 2H, H_3_, H_7_), 4.24 (br, 2H, H_10_), 3.04-2.94 (m, 2H, H_11_), 1.63-1.53 (m, 2H, H_12_), 1.31-1.12 (m, 4H, H_13_, H_14_); ^1^H NMR (400 MHz, [D_6_]DMSO, 298 K) *δ*: *integration given per ‘half’ of the guest* 9.24 (s, 1H, H_5_), 8.09 (d, *J* = 8.4 Hz, 1H, H_4_), 7.86 (dd, *J* = 8.1 Hz, 1.7 Hz, 1H, H_6_), 7.45 (ddd, *J* = 8.1 Hz, 6.9 Hz, 1.7 Hz, 1H, H_8_), 7.05-6.99 (m, H_2_, 2H, H_9_), 6.92 (dd, *J* = 8.4 Hz, 1.1 Hz, 1H, H_1_), 6.73 (ddd, *J* = 8.1 Hz, 6.9 Hz, 1.2 Hz, 1H, H_7_), 6.62 (ddd, *J* = 8.4 Hz, 7.0 Hz, 1.1 Hz, 1H, H_3_), 5.51 (br, 2H, H_10_), 2.84-2.74 (m, 2H, H_11_), 1.77-1.67 (m, 2H, H_12_), 1.40-1.26 (m, 4H, H_13_, H_14_); ^13^C NMR (100 MHz, CDCl_3_, 298 K) *δ*: 167.8, 161.7, 142.2, 140.0, 134.1, 132.6, 128.7, 122.4, 121.4, 118.0, 116.4, 116.0, 115.1, 46.4, 31.1, 28.8, 26.3; *D* (× 10^-10^ m^2^ s^-1^, 400 MHz, [D_6_]DMSO, 298 K) = 1.41; HR ESI-MS (DMF) *m/z* = 957.2218 [**G_2Pt_** + H^+^]^+^ (calc. for C_34_H_38_ N_4_O_4_Pt_2_^+^, 957.2261); IR *v* (cm^-1^) 3295, 3278, 3184, 3115, 3062, 2922, 2848, 1596, 1579, 1525, 1474, 1432.

Figure S9 ^1^H NMR spectrum (400 MHz, CDCl_3_, 298 K) of G_2Pt_.

Figure S10 ^1^H NMR spectrum (400 MHz, [D_6_]DMSO, 298 K) of G_2Pt_.

 Figure S11 ^13^C NMR spectrum (100 MHz, CDCl_3_, 298 K) of G_2Pt_.

Figure S12 ^1^H DOSY NMR spectrum (400 MHz, [D_6_]DMSO, 298 K) of G_2Pt_.

### 1.3.4. G_py_

2-Methoxyethyl 4-methylbenzenesulfonate (0.120 g, 0.521 mmol), pyrene-1-ol (0.137 g, 0.625 mmol) and potassium carbonate (0.094 g, 0.68 mmol) were dissolved in DMF (15 mL) and the mixture was heated at 80 °C overnight. The reaction mixture was diluted with DCM (100 mL) and the organic layer was washed with water (5 × 100 mL). The solvents were removed under reduced pressure. The residue was purified by column chromatography (eluting with DCM) to give the desired product as a golden oil after removal of solvent. Yield: 0.110 g (76%).

^1^H NMR (400 MHz, CDCl_3_, 298 K) δ: 8.55 (1H, d, *J* = 9.2 Hz, H_2_), 8.17-7.90 (7H, m, H_1_, H_3_, H_4_, H_5_, H_6_, H_7_, H_8_), 7.46 (1H, d, *J* = 8.4 Hz, H_9_), 4.39 (2H, m, H_10_), 3.91 (2H, m, H_11_), 3.57 (3H, s, H_12_); ^13^C NMR (100 MHz, CDCl_3_, 298 K) δ: 152.9, 131.74, 131.71, 127.3, 126.4, 126.1, 125.8, 125.5, 125.1, 124.9, 124.3, 124.2, 121.4, 120.6, 109.4, 71.3, 68.5, 59.4; HR ESI-MS (methanol) *m/z* = 277.1246 [**G_py_** + H^+^]^+^ (calc. for C_19_H_17_O_2_^+^, 277.1223); IR *v* (cm^-1^) 3041, 2983, 2925, 2876, 2823, 1628, 1599, 1507, 1488, 1452, 1433, 1407.

Figure S13 ^1^H NMR spectrum (400 MHz, CDCl_3_, 298 K) of G_py_.

Figure S14 ^13^C NMR spectrum (100 MHz, CDCl_3_, 298 K) of G_py_.

## 1.4. Complexes

### 1.4.1. [Pd(AD-Bz)_2_](BF_4_)_2_

[Pd(CH_3_CN)_4_](BF_4_)_2_ (37 mg, 0.083 mmol) and **AD-Bz** (49 mg, 0.17 mmol) were combined in DMSO (650 μL) at room temperature. The solution was added to ethyl acetate (9 mL) and the resulting precipitate was collected by centrifugation (4,000 RPM, 20 minutes), then washed with DCM (5 mL) and diethyl ether (5 mL), then dried under vacuum to give the product as a white solid (68 mg, 0.075 mmol, 90%). ^1^H NMR (400 MHz, [D_6_]DMSO, 298 K) *δ*: *integration given per ‘half’ of the complex* 9.48-9.46 (2H, m, H_a_, H_e_), 8.53 (1H, ddd, *J* = 8.0 Hz, 7.7 Hz, 1.4 Hz, H_c_), 8.47 (1H, dd, *J* = 8.0 Hz, 1.4 Hz, H_d_), 7.93 (1H, ddd, *J* = 6.7 Hz, 5.8 Hz, 1.4 Hz, H_b_), 6.75 (2H, d, *J* = 2.3 Hz, H_g_), 6.58 (1H, t, *J* = 2.3 Hz, H_h_), 5.95 (2H, s, H_f_), 3.77 (6H, s, H_i_); *D* (× 10^- 10^ m^2^ s^-1^, 400 MHz, [D_6_]DMSO, 298 K) = 1.22; HR ESI-MS (DMSO/acetonitrile) *m/z* = 349.0779 **[Pd(AD‑Bz)_2_]^2+^** (calc. for C_32_H_32_N_8_O_4_Pd^2+^, 349.0801), *m/z* = 717.1586 **[Pd(AD-Bz)_2_ + F^-^]^+^** (calc. for C_32_H_32_N_8_O_4_PdF^+^, 717.1552), *m/z* = 785.1590 **[Pd(AD-Bz)_2_ + BF_4_^-^]^+^** (calc. for C_32_H_32_N_8_O_4_PdBF_4_^+^, 785.1498).

Figure S15 ^1^H NMR spectrum (400 MHz, [D_6_]DMSO, 298 K) of [Pd(AD-Bz)_2_](BF_4_)_2_.

Figure S16 Partial ^1^H NMR spectra (400 MHz, [D_6_]DMSO, 298 K) of a) AD-Bz and b) [Pd(AD-Bz)_2_](BF_4_)_2_.

Figure S17 ^1^H DOSY NMR spectrum (400 MHz, [D_6_]DMSO, 298 K) of [Pd(AD-Bz)_2_](BF_4_)_2_.

Figure S18 HR-ESI MS (DMSO/acetonitrile) of [Pd(AD-Bz)_2_](BF_4_)_2_ and related adducts and fragments.

### 1.4.2. [Pt(AD-Bz)_2_](BF_4_)_2_

A combination of [Pt(DMSO)_2_Cl_2_] (35 mg, 0.083 mmol) and AgBF_4_ (33 mg, 0.17 mmol) in DMSO (650 μL) was heated at 50 °C in the absence of light for 20 minutes. After centrifugation (6,000 RPM, 20 minutes) the supernatant was added to **AD-Bz** (61 mg, 0.21 mmol) and heated at 50 °C for 4 hours. After centrifugation (6,000 RPM, 20 minutes), the pellet was discarded and the supernatant was added to ethyl acetate (9 mL). The precipitate was collected by centrifugation (6,000 RPM, 45 minutes), then washed with DCM (5 mL) and diethyl ether (5 mL), then dried under vacuum to give the product as a white solid (71 mg, 0.070 mmol, 85%). ^1^H NMR (400 MHz, [D_6_]DMSO, 298 K) *δ*: *integration given per ‘half’ of the complex* 9.83 (1H, d, *J* = 5.9 Hz, H_a_), 9.47 (1H, s, H_e_), 8.58 (1H, ddd, *J* = 8.0 Hz, 7.0 Hz, 1.4 Hz, H_c_), 8.52 (1H, dd, *J* = 8.0 Hz, 1.4 Hz, H_d_), 7.95 (1H, ddd, *J* = 7.0 Hz, 5.9 Hz, 1.4 Hz, H_b_), 6.79 (2H, d, *J* = 2.3 Hz, H_g_), 6.58 (1H, t, *J* = 2.3 Hz, H_h_), 5.97 (2H, s, H_f_), 3.77 (6H, s, H_i_); *D* (× 10^-10^ m^2^ s^-1^, 400 MHz, [D_6_]DMSO, 298 K) = 1.20; HR ESI-MS (DMSO/acetonitrile) *m/z* = 393.6044 **[Pt(AD-Bz)_2_]^2+^** (calc. for C_32_H_32_N_8_O_4_Pt^2+^, 393.6078), *m/z* = 806.2119 **[Pt(AD-Bz)_2_ + F^-^]^+^** (calc. for C_32_H_32_N_8_O_4_PtF^+^, 806.2219), *m/z* = 874.2180 **[Pt(AD-Bz)_2_ + BF_4_^-^]^+^** (calc. for C_32_H_32_N_8_O_4_PtBF_4_^+^, 874.2222).

Figure S19 ^1^H NMR spectrum (400 MHz, [D_6_]DMSO, 298 K) of [Pt(AD-Bz)_2_](BF_4_)_2_.

Figure S20 Partial ^1^H NMR spectra (400 MHz, [D_6_]DMSO, 298 K) of a) AD-Bz and b) [Pt(AD-Bz)_2_](BF_4_)_2_.

Figure S21 ^1^H DOSY NMR spectrum (400 MHz, [D_6_]DMSO, 298 K) of [Pt(AD-Bz)_2_](BF_4_)_2_.

Figure S22 HR-ESI MS (DMSO/acetonitrile) of [Pt(AD-Bz)_2_](BF_4_)_2_ and related adducts and fragments.

### 1.4.3. [Pt(AD-PEG)_2_](BF_4_)_2_

*Note: this complex is only used in crystallisations.*

A combination of [Pt(DMSO)_2_Cl_2_] (10.0 mg, 0.0237 mmol) and AgBF_4_ (9.45 mg, 0.0485 mmol) in DMSO (200 μL) was heated at 50 °C in the absence of light for 20 minutes. After centrifugation (6,000 RPM, 20 minutes) the supernatant was added to **AD-PEG**^[2]^ (12.1 mg, 0.0592 mmol) and heated at 50 °C overnight. After centrifugation (6,000 RPM, 20 minutes), the pellet was discarded and the supernatant was added to ethyl acetate (1 mL). The precipitate was collected by centrifugation (6,000 RPM, 45 minutes), then washed with DCM (0.5 mL) and diethyl ether (0.5 mL), then dried under vacuum to give the product as a white solid (11 mg, 0.054 mmol, 91%). ^1^H NMR (400 MHz, CD_3_CN, 298 K) *δ*: *integration given per ‘half’ of the complex* 9.88 (1H, d, *J* = 6.0 Hz, H_a_), 8.87 (1H, s, H_e_), 8.44 (1H, ddd, *J* = 8.3 Hz, 7.8 Hz, 1.4 Hz, H_c_), 8.28 (1H, dd, *J* = 7.8 Hz, 1.4 Hz, H_d_), 7.84 (1H, ddd, *J* = 7.8 Hz, 6.0 Hz, 1.4 Hz, H_b_), 4.91 (2H, t, *J* = 4.9 Hz, H_f_), 3.96 (2H, t, *J* = 4.9 Hz, H_g_), 3.40 (3H, s, H_h_); *D* (× 10^-10^ m^2^ s^-1^, 400 MHz, CD_3_CN, 298 K) = 0.96; HR ESI-MS (acetonitrile) *m/z* = 301.5890 **[Pt(AD-PEG)_2_]^2+^** (calc. for C_20_H_24_N_8_O_2_Pt^2+^, 301.5836), *m/z* = 622.1655 **[Pt(AD‑PEG)_2_ + F^-^]^+^** (calc. for C_20_H_24_N_8_O_2_PtF^+^, 622.1655), *m/z* = 690.1701 **[Pt(AD-PEG)_2_ + BF_4_^-^]^+^** (calc. for C_20_H_24_N_8_O_2_PtBF_4_^+^, 690.1708).

Figure S23 ^1^H NMR spectrum (400 MHz, CD_3_CN, 298 K) of [Pt(AD-PEG)_2_](BF_4_)_2_.

Figure S24 ^1^H DOSY NMR spectrum (400 MHz, CD_3_CN, 298 K) of [Pt(AD-PEG)_2_](BF_4_)_2_.

Figure S25 HR-ESI MS (acetonitrile) of [Pt(AD-PEG)_2_](BF_4_)_2_ and related adducts and fragments.

# 2. Host-Guest Chemistry

## 2.1. Mass spectrometry

Figure S26 HR-ESI MS (DMSO/acetonitrile) of [G_2py_ ⊂ Pd(AD-Bz)_2_](BF_4_)_2_ and related adducts and fragments.

Figure S27 HR-ESI MS (DMSO/acetonitrile) of [G_2py_ ⊂ Pt(AD-Bz)_2_](BF_4_)_2_ and related adducts and fragments.

Figure S28 HR-ESI MS (DMSO/acetonitrile) of [G_2nap_ ⊂ Pd(AD-Bz)_2_](BF_4_)_2_ and related adducts and fragments.

Figure S29 HR-ESI MS (DMSO/acetonitrile) of [G_2nap_ ⊂ Pt(AD-Bz)_2_](BF_4_)_2_ and related adducts and fragments.

Figure S30 HR-ESI MS (DMSO/acetonitrile) of [G_2Pt_ ⊂ Pd(AD-Bz)_2_](BF_4_)_2_ and related adducts and fragments.

Figure S31 HR-ESI MS (DMSO/acetonitrile) of [G_2Pt_ ⊂ Pt(AD-Bz)_2_](BF_4_)_2_ and related adducts and fragments.

## 2.2. NMR data

### 2.2.1. [G_2py_ ⊂ Pd(AD-Bz)_2_](BF_4_)_2_

Figure S32 Partial ^1^H NMR spectra (400 MHz, [D_6_]DMSO, 298 K) of a) [Pd(AD-Bz)_2_](BF_4_)_2_, b) G_2py_ (1 eq.) + [Pd(AD-Bz)_2_](BF_4_)_2_ and c) G_2py_.

Figure S33 ^1^H DOSY NMR spectrum (400 MHz, [D_6_]DMSO, 298 K) of [G_2py_ ⊂ Pd(AD-Bz)_2_](BF_4_)_2_.

### 2.2.2. [G_2py_ ⊂ Pt(AD-Bz)_2_](BF_4_)_2_

Figure S34 Partial ^1^H NMR spectra (400 MHz, [D_6_]DMSO, 298 K) of a) [Pt(AD-Bz)_2_](BF_4_)_2_, b) G_2py_ (1 eq.) + [Pt(AD-Bz)_2_](BF_4_)_2_ and c) G_2py_.

Figure S35 ^1^H DOSY NMR spectrum (400 MHz, [D_6_]DMSO, 298 K) of [G_2py_ ⊂ Pt(AD-Bz)_2_](BF_4_)_2_.

### 2.2.3. [G_2nap_ ⊂ Pd(AD-Bz)_2_](BF_4_)_2_

Figure S36 Partial ^1^H NMR spectra (400 MHz, [D_6_]DMSO, 298 K) of a) [Pd(AD-Bz)_2_](BF_4_)_2_, b) G_2nap_ (1 eq.) + [Pd(AD-Bz)_2_](BF_4_)_2_ and c) G_2nap_.

Figure S37 ^1^H DOSY NMR spectrum (400 MHz, [D_6_]DMSO, 298 K) of [G_2nap_ + Pd(AD-Bz)_2_](BF_4_)_2_.

### 2.2.4. [G_2nap_ ⊂ Pt(AD-Bz)_2_](BF_4_)_2_

Figure S38 Partial ^1^H NMR spectra (400 MHz, [D_6_]DMSO, 298 K) of a) [Pt(AD-Bz)_2_](BF_4_)_2_, b) G_2nap_ (1 eq.) + [Pt(AD-Bz)_2_](BF_4_)_2_ and c) G_2nap_.

Figure S39 ^1^H DOSY NMR spectrum (400 MHz, [D_6_]DMSO, 298 K) of [G_2nap_ + Pt(AD-Bz)_2_](BF_4_)_2_.

### 2.2.5. [G_2Pt_ ⊂ Pd(AD-Bz)_2_](BF_4_)_2_

Figure S40 Partial ^1^H NMR spectra (400 MHz, [D_6_]DMSO, 298 K) of a) [Pd(AD-Bz)_2_](BF_4_)_2_, b) G_2Pt_ (1 eq.) + [Pd(AD-Bz)_2_](BF_4_)_2_ and c) G_2Pt_.

Figure S41 ^1^H DOSY NMR spectrum (400 MHz, [D_6_]DMSO, 298 K) of [G_2Pt_ ⊂ Pd(AD-Bz)_2_](BF_4_)_2_.

### 2.2.6. [G_2Pt_ ⊂ Pt(AD-Bz)_2_](BF_4_)_2_

Figure S42 Partial ^1^H NMR spectra (400 MHz, [D_6_]DMSO, 298 K) of a) [Pt(AD-Bz)_2_](BF_4_)_2_, b) G_2Pt_ (1 eq.) + [Pt(AD-Bz)_2_](BF_4_)_2_ and c) G_2Pt_.

Figure S43 ^1^H DOSY NMR spectrum (400 MHz, [D_6_]DMSO, 298 K) of [G_2Pt_ ⊂ Pt(AD-Bz)_2_](BF_4_)_2_.

## 2.3. ^1^H DOSY NMR Summary

The DOSY coefficients for each of the host-guest complexes is in line with those of the hosts and guests, supporting the notion that host-guest complexes are formed as described: a single guest wraps around the face of the host, to give a 1:1 host-guest complex.

Table S1 Summary of 1H DOSY NMR coefficients of the organic compounds and complexes.

Table S2 Summary of 1H DOSY NMR coefficients of the host-guest complexes.

## 2.4. NMR titration data

### 2.4.1. General titration information

Solutions of **[M(AD-Bz)_2_](BF_4_)_2_** (either **[Pd(AD-Bz)_2_](BF_4_)_2_** or **[Pt(AD-Bz)_2_](BF_4_)_2_)** at 1mM in [D_6_]DMSO were treated with aliquots of equal volume of **[M(AD-Bz)_2_](BF_4_)_2_** and the guest (in the same solvent), such that the overall concentration of **[M(AD-Bz)_2_](BF_4_)_2_** remained constant and the concentration of guest increased with each aliquot. The equivalencies were checked via integration of the ^1^H NMR spectra. Titrations were continued until at least 10 equivalences of guest were present. Where binding was weak and titration endpoints not obvious at 10 equivalences, the titrations were repeated in the less competitive solvent, CD_3_CN. Poor solubility of the guest **G_2Pt_** precluded repeating this titration in CD_3_CN.

Binding constants were calculated in each case using Bindfit^[10-12]^ across all models of 1:1, 1:2 and 2:1 host/guest stoichiometry. It is expected that primarily due to entropic reasons, binding likely occurs in a 1:1 model. In most cases the errors associated or binding constant values themselves obtained through 1:2 and 2:1 binding modes support the notion that 1:1 binding predominates, as detailed below. It is important to note that for weakly binding systems, the lack of an obvious endpoint or significant changes in chemical shifts can result in data that fits to multiple binding modes with little error. Constants were calculated from the chemical shift of all completely unobscured protons of the host in each case.

Figure S44 Labelled Chemdraw structures of [Pd(AD-Bz)_2_](BF_4_)_2_ and [Pt(AD-Bz)_2_](BF_4_)_2_ for reference.

### 2.4.2. [G_2py_ ⊂ Pd(AD-Bz)_2_](BF_4_)_2_

Figure S45 Stacked partial ^1^H NMR spectra (400 MHz, [D_6_]DMSO, 298 K) highlighting the shifts of unobscured proton environments of [Pd(AD-Bz)_2_](BF_4_)_2_ as increasing equivalents of G_2py_ were added (equivalences indicated on spectra).

Figure S46 Demonstrates the change in chemical shifts of unobscured proton environments of [Pd(AD-Bz)_2_](BF_4_)_2_: H_e_ (blue), H_g_ (grey), H_h_ (red) and H_f_ (green), as increasing equivalents of G_2py_ were added in [D_6_]DMSO. Data points depict the raw data and trendlines depict the fit provided by the Bindfit program using a 1:1 binding model.

Figure S47 Demonstrates the chemical shift residuals between the obtained data points and the fit provided by the Bindfit program using a 1:1 binding model of each unobscured proton environment of [Pd(AD-Bz)_2_](BF_4_)_2_: H_e_ (blue), H_g_ (grey), H_h_ (red) and H_f_ (green), as increasing equivalents of G_2py_ were added in [D_6_]DMSO.

Figure S48 Relationship between the RMSE (ppm) and the value of *K*_1_ for a 1:1 binding model between G_2py_ and [Pd(AD‑Bz)_2_](BF_4_)_2_ in [D_6_]DMSO fitted using Musketeer.

Table S3 Details the binding constants and errors (percentage error, root mean squared (RMS) and co-variance) of the calculated fit provided by the Bindfit program for each binding mode on the data collected from titrating G_2py_ into [Pd(AD‑Bz)_2_](BF_4_)_2_ in [D_6_]DMSO. Extremely large errors and/or negative (impossible) values are highlighted with red text.

### 2.4.3. [G_2py_ ⊂ Pt(AD-Bz)_2_](BF_4_)_2_

Figure S49 Stacked partial ^1^H NMR spectra (400 MHz, [D_6_]DMSO, 298 K) highlighting the shifts of unobscured proton environments of [Pt(AD-Bz)_2_](BF_4_)_2_ as increasing equivalents of G_2py_ were added (equivalences indicated on spectra).

Figure S50 Demonstrates the change in chemical shifts of unobscured proton environments of [Pt(AD-Bz)_2_](BF_4_)_2_: H_e_ (blue), H_g_ (grey), H_h_ (red) and H_f_ (green), as increasing equivalents of G_2py_ were added in [D_6_]DMSO. Data points depict the raw data and trendlines depict the fit provided by the Bindfit program.

Figure S51 Demonstrates the chemical shift residuals between the obtained data points and the fit provided by the Bindfit program using a 1:1 binding model of each unobscured proton environment of [Pt(AD-Bz)_2_](BF_4_)_2_: H_e_ (blue), H_g_ (grey), H_h_ (red) and H_f_ (green), as increasing equivalents of G_2py_ were added in [D_6_]DMSO.

Figure S52 Relationship between the RMSE (ppm) and the value of *K*_1_ for a 1:1 binding model between G_2py_ and [Pt(AD‑Bz)_2_](BF_4_)_2_ in [D_6_]DMSO fitted using Musketeer.

Table S4 Details the binding constants and errors (percentage error, root mean squared (RMS) and co-variance) of the calculated fit provided by the Bindfit program for each binding mode on the data collected from titrating G_2py_ into [Pt(AD‑Bz)_2_](BF_4_)_2_ in [D_6_]DMSO. Extremely large errors and/or negative (impossible) values are highlighted with red text.

### 2.4.4. [G_2nap_ ⊂ Pd(AD-Bz)_2_](BF_4_)_2_

Figure S53 Stacked partial ^1^H NMR spectra (400 MHz, [D_6_]DMSO, 298 K) highlighting the shifts of unobscured proton environments of [Pd(AD-Bz)_2_](BF_4_)_2_ as increasing equivalents of G_2nap_ were added (equivalences indicated on spectra).

Figure S54 Demonstrates the change in chemical shifts of unobscured proton environments of [Pd(AD-Bz)_2_](BF_4_)_2_: H_e_ (grey), H_a_ (blue), H_g_ (red), H_h_ (green) and H_f_ (orange), as increasing equivalents of G_2nap_ were added in [D_6_]DMSO. Data points depict the raw data and trendlines depict the fit provided by the Bindfit program.

Table S5 Details the binding constants and errors (percentage error, root mean squared (RMS) and co-variance) of the calculated fit provided by the Bindfit program for each binding mode on the data collected from titrating G_2nap_ into [Pd(AD‑Bz)_2_](BF_4_)_2_ in [D_6_]DMSO. Extremely large errors and/or negative (impossible) values are highlighted with red text.

Figure S55 Stacked partial ^1^H NMR spectra (400 MHz, CD_3_CN, 298 K) highlighting the shifts of unobscured proton environments of [Pd(AD-Bz)_2_](BF_4_)_2_ as increasing equivalents of G_2nap_ were added (equivalences indicated on spectra).

Figure S56 Demonstrates the change in chemical shifts of unobscured proton environments of [Pd(AD-Bz)_2_](BF_4_)_2_: H_e_ (grey), H_a_ (blue), H_c_ (red) and H_d_ (green), as increasing equivalents of G_2nap_ were added in CD_3_CN. Data points depict the raw data and trendlines depict the fit provided by the Bindfit program.

Table S6 Details the binding constants and errors (percentage error, root mean squared (RMS) and co-variance) of the calculated fit provided by the Bindfit program for each binding mode on the data collected from titrating G_2nap_ into [Pd(AD‑Bz)_2_](BF_4_)_2_ in CD_3_CN. Extremely large errors and/or negative (impossible) values are highlighted with red text.

### 2.4.5. [G_2nap_ ⊂ Pt(AD-Bz)_2_](BF_4_)_2_

Figure S57 Stacked partial ^1^H NMR spectra (400 MHz, [D_6_]DMSO, 298 K) highlighting the shifts of unobscured proton environments of [Pt(AD-Bz)_2_](BF_4_)_2_ as increasing equivalents of G_2nap_ were added (equivalences indicated on spectra).

Figure S58 Demonstrates the change in chemical shifts of unobscured proton environments of [Pt(AD-Bz)_2_](BF_4_)_2_: H_a_ (grey), H_e_ (blue), H_g_ (red), H_h_ (green) and H_f_ (orange) as increasing equivalents of G_2nap_ were added in [D_6_]DMSO. Data points depict the raw data and trendlines depict the fit provided by the Bindfit program.

Table S7 Details the binding constants and errors (percentage error, root mean squared (RMS) and co-variance) of the calculated fit provided by the Bindfit program for each binding mode on the data collected from titrating G_2nap_ into [Pt(AD‑Bz)_2_](BF_4_)_2_ in [D_6_]DMSO. Extremely large errors and/or negative (impossible) values are highlighted with red text.

Figure S59 Stacked partial ^1^H NMR spectra (400 MHz, CD_3_CN, 298 K) highlighting the shifts of unobscured proton environments of [Pt(AD-Bz)_2_](BF_4_)_2_ as increasing equivalents of G_2nap_ were added (equivalences indicated on spectra).

Figure S60 Demonstrates the change in chemical shifts of unobscured proton environments of [Pt(AD-Bz)_2_](BF_4_)_2_: H_a_ (grey), H_e_ (blue), H_c_ (red) and H_d_ (green) as increasing equivalents of G_2nap_ were added in CD_3_CN. Data points depict the raw data and trendlines depict the fit provided by the Bindfit program.

Table S8 Details the binding constants and errors (percentage error, root mean squared (RMS) and co-variance) of the calculated fit provided by the Bindfit program for each binding mode on the data collected from titrating G_2nap_ into [Pt(AD‑Bz)_2_](BF_4_)_2_ in CD_3_CN. Extremely large errors and/or negative (impossible) values are highlighted with red text.

### 2.4.6. [G_2Pt_ ⊂ Pd(AD-Bz)_2_](BF_4_)_2_

Figure S61 Stacked partial ^1^H NMR spectra (400 MHz, [D_6_]DMSO, 298 K) highlighting the shifts of unobscured proton environments of [Pd(AD-Bz)_2_](BF_4_)_2_ as increasing equivalents of G_2Pt_ were added (equivalences indicated on spectra).

Figure S62 Demonstrates the change in chemical shifts of unobscured proton environments of [Pd(AD-Bz)_2_](BF_4_)_2_: H_e_ (grey), H_a_ (blue), H_c_ (red) and H_d_ (green) as increasing equivalents of G_2Pt_ were added in [D_6_]DMSO. Data points depict the raw data and trendlines depict the fit provided by the Bindfit program.

Figure S63 Demonstrates the chemical shift residuals between the obtained data points and the fit provided by the Bindfit program using a 1:1 binding model of each unobscured proton environment of [Pd(AD-Bz)_2_](BF_4_)_2_: H_a_ (blue), H_e_ (grey), H_c_ (red) and H_d_ (green), as increasing equivalents of G_2Pt_ were added in [D_6_]DMSO.

Figure S64 Relationship between the RMSE (ppm) and the value of *K*_1_ for a 1:1 binding model between G_2Pt_ and [Pd(AD‑Bz)_2_](BF_4_)_2_ in [D_6_]DMSO fitted using Musketeer.

Table S9 Details the binding constants and errors (percentage error, root mean squared (RMS) and co-variance) of the calculated fit provided by the Bindfit program for each binding mode on the data collected from titrating G_2Pt_ into [Pd(AD‑Bz)_2_](BF_4_)_2_ in [D_6_]DMSO. Extremely large errors and/or negative (impossible) values are highlighted with red text.

### 2.4.7. [G_2Pt_ ⊂ Pt(AD-Bz)_2_](BF_4_)_2_

Figure S65 Stacked partial ^1^H NMR spectra (400 MHz, [D_6_]DMSO, 298 K) highlighting the shifts of unobscured proton environments of [Pt(AD-Bz)_2_](BF_4_)_2_ as increasing equivalents of G_2Pt_ were added (equivalences indicated on spectra).

Figure S66 Demonstrates the change in chemical shifts of unobscured proton environments of [Pt(AD-Bz)_2_](BF_4_)_2_: H_a_ (grey), H_e_ (blue), H_c_ (red) and H_d_ (green) as increasing equivalents of G_2Pt_ were added in [D_6_]DMSO. Data points depict the raw data and trendlines depict the fit provided by the Bindfit program.

Figure S67 Demonstrates the chemical shift residuals between the obtained data points and the fit provided by the Bindfit program using a 1:1 binding model of each unobscured proton environment of [Pt(AD-Bz)_2_](BF_4_)_2_: H_e_ (blue), H_a_ (grey), H_c_ (red) and H_d_ (green), as increasing equivalents of G_2Pt_ were added in [D_6_]DMSO.

Figure S68 Relationship between the RMSE (ppm) and the value of *K*_1_ for a 1:1 binding model between G_2Pt_ and [Pt(AD‑Bz)_2_](BF_4_)_2_ in [D_6_]DMSO fitted using Musketeer.

Table S10 Details the binding constants and errors (percentage error, root mean squared (RMS) and co-variance) of the calculated fit provided by the Bindfit program for each binding mode on the data collected from titrating G_2Pt_ into [Pt(AD‑Bz)_2_](BF_4_)_2_ in [D_6_]DMSO. Extremely large errors and/or negative (impossible) values are highlighted with red text.

### 2.4.8. [G_py_ ⊂ Pd(AD-Bz)_2_](BF_4_)_2_

Figure S69 Stacked partial ^1^H NMR spectra (400 MHz, CD_3_CN, 298 K) highlighting the shifts of unobscured proton environments of [Pd(AD-Bz)_2_](BF_4_)_2_ as increasing equivalents of G_py_ were added (equivalences indicated on spectra). Precipitation of G_py_ due to poor solubility precluded further addition of the guest.

Figure S70 Demonstrates the change in chemical shifts of unobscured proton environments in [Pd(AD-Bz)_2_](BF_4_)_2_: H_e_ (blue), H_g_ (red), H_h_ (green) and H_f_ (orange) as increasing equivalents of G_py_ were added in CD_3_CN. Data points depict the raw data and trendlines depict the fit provided by the Bindfit program.

Figure S71 Demonstrates the chemical shift residuals between the obtained data points and the fit provided by the Bindfit program using a 1:1 binding model of each unobscured proton environment of [Pd(AD-Bz)_2_](BF_4_)_2_: H_e_ (blue), H_g_ (red), H_h_ (green) and H_f_ (orange) as increasing equivalents of G_py_ were added in CD_3_CN.

Table S11 Details the binding constants and errors (percentage error, root mean squared (RMS) and co-variance) of the calculated fit provided by the Bindfit program for each binding mode on the data collected from titrating G_py_ into [Pd(AD‑Bz)_2_](BF_4_)_2_ in CD_3_CN. Extremely large errors and/or negative (impossible) values are highlighted with red text.

### 2.4.9. [G_py_ ⊂ Pt(AD-Bz)_2_](BF_4_)_2_

Figure S72 Stacked partial ^1^H NMR spectra (400 MHz, CD_3_CN, 298 K) highlighting the shifts of unobscured proton environments of [Pt(AD-Bz)_2_](BF_4_)_2_ as increasing equivalents of G_py_ were added (equivalences indicated on spectra).

Figure S73 Demonstrates the change in chemical shifts of unobscured proton environments of [Pt(AD-Bz)_2_](BF_4_)_2_: H_e_ (blue), H_g_ (red), H_h_ (green) and H_f_ (orange) as increasing equivalents of G_py_ were added in CD_3_CN. Data points depict the raw data and trendlines depict the fit provided by the Bindfit program.

Figure S74 Demonstrates the chemical shift residuals between the obtained data points and the fit provided by the Bindfit program using a 1:1 binding model of each unobscured proton environment of [Pt(AD-Bz)_2_](BF_4_)_2_: H_e_ (blue), H_g_ (red), H_h_ (green) and H_f_ (orange), as increasing equivalents of G_py_ were added in CD_3_CN.

Table S12 Details the binding constants and errors (percentage error, root mean squared (RMS) and co-variance) of the calculated fit provided by the Bindfit program for each binding mode on the data collected from titrating G_py_ into [Pt(AD‑Bz)_2_](BF_4_)_2_ in CD_3_CN. Extremely large errors and/or negative (impossible) values are highlighted with red text.

*Note*: A 1:1 binding model was used for reporting of binding constants for **[G_py_ ⊂ Pd(AD-Bz)_2_](BF_4_)_2_** and **[G_py_ ⊂ Pt(AD-Bz)_2_](BF_4_)_2_** as the second binding event is likely too weak to be observed in the limited [G]/[H] equivalences reached due to the poor solubility of **G_py_**.

## 2.5. Calculation of Binding Constants (1:1 Model)

### 2.5.1. General Information

The binding constants and associated errors calculated using Bindfit combined with basic experimental observations (e.g. guest equivalences) it was determined that a 1:1 binding mode is preferred. To ensure the differences observed in calculated binding constants were accurate, binding constants and the program’s chosen measures of error were additionally calculated using Bindtools^[13]^ and Musketeer.^[14]^ Relatively low error is observed across the 1:1 models of all host-guest combinations from multiple programs.

### 2.5.2. G_2py_

Table S13 Details the binding constants and errors of the calculated fit provided by the Bindfit program, Bindtools and Musketeer for each binding mode on the data collected from titrating G_2py_ into [Pd(AD-Bz)_2_](BF_4_)_2_ and [Pt(AD-Bz)_2_](BF_4_)_2_ in [D_6_]DMSO.

### 2.5.3. G_2nap_

Table S14 Details the binding constants and errors of the calculated fit provided by the Bindfit program, Bindtools and Musketeer for each binding mode on the data collected from titrating G_2nap_ into [Pd(AD-Bz)_2_](BF_4_)_2_ and [Pt(AD-Bz)_2_](BF_4_)_2_ in CD_3_CN.

Table S15 Details the binding constants and errors of the calculated fit provided by the Bindfit program, Bindtools and Musketeer for each binding mode on the data collected from titrating G_2nap_ into [Pd(AD-Bz)_2_](BF_4_)_2_ and [Pt(AD-Bz)_2_](BF_4_)_2_ in [D_6_]DMSO.

### 2.5.4. G_2Pt_

Table S16 Details the binding constants and errors of the calculated fit provided by the Bindfit program, Bindtools and Musketeer for each binding mode on the data collected from titrating G_2Pt_ into [Pd(AD-Bz)_2_](BF_4_)_2_ and [Pt(AD-Bz)_2_](BF_4_)_2_ in [D_6_]DMSO.

## 2.6. Competition Experiments

### 2.6.1. General Information

Stock solutions of **[Pd(AD-Bz)_2_](BF_4_)_2_** and **[Pt(AD-Bz)_2_](BF_4_)_2_** in [D_6_]DMSO or CD_3_CN were combined in a 1:1 ratio such that each competition experiment began with as a 550 μL NMR-scale mixture with each complex at a concentration of 3 mM. Each mixture was treated with aliquots of the respective guest from a concentrated stock solution until either 2.00 eq. (if full titration data was available for the guest and solvent combination) or an excess of guest was present (if titrations were not possible under the conditions). The equivalencies were checked via integration of the ^1^H NMR spectra.

### 2.6.2. G_2py_

Figure S75 Stacked partial ^1^H NMR spectra (400 MHz, [D_6_]DMSO, 298 K) highlighting the shifts of H_e_ in [Pd(AD-Bz)_2_](BF_4_)_2_ (blue) and [Pt(AD-Bz)_2_](BF_4_)_2_ (red) as increasing equivalents of G_2py_ were added (equivalences indicated on spectra).

Figure S76 Demonstrates the change in host-guest (HG) molefraction of [Pd(AD-Bz)_2_](BF_4_)_2_ (blue) and [Pt(AD-Bz)_2_](BF_4_)_2_ (red) as increasing equivalents of G_2py_ were added in [D_6_]DMSO. HG molefractions have been calculated based on the chemical shift of H_a_ relative to the HG chemical shift predicted by Bindfit.

Table S17 Details the change in chemical shift of H_a_ of [Pd(AD-Bz)_2_](BF_4_)_2_ and [Pt(AD-Bz)_2_](BF_4_)_2_ in the same solution of [D_6_]DMSO between the free host (H), varied guest-host ratios ([G_2py_]/[H]) and the HG_2py_ complex as predicted by Bindfit.

Figure S77 Stacked partial ^1^H NMR spectra (400 MHz, CD_3_CN, 298 K) highlighting the shifts of H_a_ in [Pd(AD-Bz)_2_](BF_4_)_2_ (blue) and [Pt(AD-Bz)_2_](BF_4_)_2_ (red) as increasing equivalents of G_2py_ were added (equivalences indicated on spectra).

Figure S78 Insets from the previous stacked partial ^1^H NMR spectra (400 MHz, CD_3_CN, 298 K) highlighting the H_a_ signals in [Pd(AD-Bz)_2_](BF_4_)_2_ which overlap with other peaks as increasing equivalents of G_2py_ were added (equivalences indicated on spectra). The spectrum at a) 0.50 eq. is present as a reference for comparison to b) where H_a_ overlaps with this signal.

Figure S79 Demonstrates the change in host-guest (HG) molefraction of [Pd(AD-Bz)_2_](BF_4_)_2_ (blue) and [Pt(AD-Bz)_2_](BF_4_)_2_ (red) as increasing equivalents of G_2py_ were added in CD_3_CN. HG molefractions have been calculated based on the chemical shift of H_a_ relative to the HG chemical shift predicted by Bindfit.

Table S18 Details the change in chemical shift of H_a_ of [Pd(AD-Bz)_2_](BF_4_)_2_ and [Pt(AD-Bz)_2_](BF_4_)_2_ in the same solution of CD_3_CN between the free host (H), varied guest-host ratios ([G_2py_]/[H]) and the HG_2py_ complex as predicted by Bindfit.

### 2.6.3. G_2nap_

Figure S80 Stacked partial ^1^H NMR spectra (400 MHz, [D_6_]DMSO, 298 K) highlighting the shifts of H_a_ in [Pd(AD-Bz)_2_](BF_4_)_2_ (blue) and [Pt(AD-Bz)_2_](BF_4_)_2_ (red) as increasing equivalents of G_2nap_ were added (equivalences indicated on spectra).

Figure S81 Demonstrates the change in host-guest (HG) molefraction of [Pd(AD-Bz)_2_](BF_4_)_2_ (blue) and [Pt(AD-Bz)_2_](BF_4_)_2_ (red) as increasing equivalents of G_2nap_ were added in [D_6_]DMSO. HG molefractions have been calculated based on the chemical shift of H_a_ relative to the HG chemical shift predicted by Bindfit.

Table S19 Details the change in chemical shift of H_a_ of [Pd(AD-Bz)_2_](BF_4_)_2_ and [Pt(AD-Bz)_2_](BF_4_)_2_ in the same solution of [D_6_]DMSO between the free host (H), varied guest-host ratios ([G_2nap_]/[H]) and the HG_2nap_ complex as predicted by Bindfit.

Figure S82 Stacked partial ^1^H NMR spectra (400 MHz, CD_3_CN, 298 K) highlighting the shifts of H_a_ in [Pd(AD-Bz)_2_](BF_4_)_2_ (blue) and [Pt(AD-Bz)_2_](BF_4_)_2_ (red) as increasing equivalents of G_2nap_ were added (equivalences indicated on spectra).

Figure S83 Demonstrates the change in host-guest (HG) molefraction of [Pd(AD-Bz)_2_](BF_4_)_2_ (blue) and [Pt(AD-Bz)_2_](BF_4_)_2_ (red) as increasing equivalents of G_2nap_ were added in CD_3_CN. HG molefractions have been calculated based on the chemical shift of H_a_ relative to the HG chemical shift predicted by Bindfit.

Table S20 Details the change in chemical shift of H_a_ of [Pd(AD-Bz)_2_](BF_4_)_2_ and [Pt(AD-Bz)_2_](BF_4_)_2_ in the same solution of CD_3_CN between the free host (H), varied guest-host ratios ([G_2nap_]/[H]) and the HG_2nap_ complex as predicted by Bindfit.

### 2.6.4. G_2Pt_

Figure S84 Stacked partial ^1^H NMR spectra (400 MHz, [D_6_]DMSO, 298 K) highlighting the shifts of H_a_ in [Pd(AD-Bz)_2_](BF_4_)_2_ (blue) and [Pt(AD-Bz)_2_](BF_4_)_2_ (red) as increasing equivalents of G_2Pt_ were added (equivalences indicated on spectra).

Figure S85 Demonstrates the change in host-guest (HG) molefraction of [Pd(AD-Bz)_2_](BF_4_)_2_ (blue) and [Pt(AD-Bz)_2_](BF_4_)_2_ (red) as increasing equivalents of G_2Pt_ were added in [D_6_]DMSO. HG molefractions have been calculated based on the chemical shift of H_a_ relative to the HG chemical shift predicted by Bindfit.

Table S21 Details the change in chemical shift of H_a_ of [Pd(AD-Bz)_2_](BF_4_)_2_ and [Pt(AD-Bz)_2_](BF_4_)_2_ in the same solution of [D_6_]DMSO between the free host (H), varied guest-host ratios ([G_2Pt_]/[H]) and the HG_2Pt_ complex as predicted by Bindfit.

# 3. Calculations

## 3.1. Structure optimisation

To investigate the differences in energy which arise from steric and electronic contributions between homoleptic, heteroleptic structures and their various isomers, idealised representations of complexes **[G_2py_ ⊂ Pd(AD‑Bz)_2_]^2+^**, **[G_2py_ ⊂ Pt(AD‑Bz)_2_]^2+^**, **[G_2nap_ ⊂ Pd(AD‑Bz)_2_]^2+^**, **[G_2nap_ ⊂ Pt(AD‑Bz)_2_]^2+^**, **[G_2Pt_ ⊂  Pd(AD-Bz)_2_]^2+^**, and **[G_2Pt_ ⊂ Pt(AD-Bz)_2_]^2+^** were first constructed in *Spartan24*,^[15]^ omitting counter ions and solubilising chains for simplicity. The further simplified hosts **[Pd(AD-model)_2_]^2+^**, **[Pt(AD-model)_2_]^2+^** and model complexes **[pyrene ⊂ Pd(AD-model)_2_]^2+^**, and **[pyrene ⊂ Pt(AD-model)_2_]^2+^** were also constructed in *Spartan24*. The positioning of the guests around the respective host and the overall conformation of the complex were informed by obtained crystal structures. These structures were then optimised using the quantum mechanical extended tight binding method GFN2-xTB 6.7.1,^[16]^ as implemented in the *xTB* code. Optimisations were performed using GFN 2, an “extreme” optimization [extreme: Econv (energy convergence) = 5 × 10^−8^ Eh; Gconv (gradient convergence) = 5 × 10^−5^ Eh∙α^−1^; accuracy (for integral cutoffs and SCF criteria) = 0.01] and the solvent DMSO was represented with an implicit solvation model (ALPB, as implemented in the *xTB* code).^[17]^ Lebedev grid level used was “normal” with 230 grid points (as implemented in the *xTB* code).^[18]^

These *xTB* optimised complexes were then optimised further with density functional theory (DFT) calculations. These were performed using the ORCA program version 6.0.1.^[19]^ To differentiate the different energetic contributions to host-guest association we chose to optimise the six complexes at increasing levels of PBE theory, working with the PBE,^[20]^ PBE0^[21]^ and PBE0D4^[21, 22]^ functionals with C and H atoms treated by the def2-SVP basis set and all other atoms  (N, O, Pd, Pt) treated by the def2-TZVP basis set.^[23]^ For the elements Pd and Pt, this basis set implements ECPs and relativistic effects.

For all calculations, the structure at the preceding level of theory was used, *ie.* to calculate **[G_2py_ ⊂ Pt(AD-Bz)_2_]^2+^** at PBE0D4, the PBE0 optimised structure of **[G_2py_ ⊂ Pt(AD-Bz)_2_]^2+^** was used. To check whether we were introducing any conformational preference by doing this, we took the PBE0D4 **[G_2py_ ⊂ M(AD-Bz)_2_]^2+^** complexes and switched the metal centres and then recalculated the energies at PBE0D4. Between the two **[G_2py_ ⊂ Pd(AD-Bz)_2_]^2+^** complexes there was an energy difference of 2.49 kJ/mol and between the two **[G_2py_ ⊂ Pt(AD-Bz)_2_]^2+^** complexes there was an energy difference of 1.64 kJ/mol.

To further probe the dynamic equilibrium of the complexes **[G_2py_ ⊂ Pd(AD-Bz)_2_]^2+^**, **[G_2py_ ⊂ Pt(AD-Bz)_2_]^2+^**, **[G_2nap_ ⊂ Pd(AD-Bz)_2_]^2+^** and **[G_2nap_ ⊂ Pt(AD‑Bz)_2_]^2+^** and possible conformers that may be more representative of our solution based binding analysis, we employed molecular dynamics (MD) simulations. All GFN2-*xTB* MD calculations were performed with initial “loose” optimization [loose: *E*conv (energy convergence) = 5×10^−5^ *E*h; *G*conv (gradient convergence) = 4×10^−3^ *E*h∙α^−1^; accuracy (for integral cutoffs and SCF criteria) = 2.00] and the solvent DMSO was represented with an implicit solvation model (ALPB, as implemented in the *xTB* code).^[17]^ Lebedev grid level used was “normal” with 230 grid points (as implemented in the *xTB* code).^[18]^ The simulations were conducted in the NVT ensemble with the system temperature being maintained at 298 K. All bonds were constrained using the SHAKE algorithm.^[24]^ Each simulation was run for 500 ps, with trajectory output every 100 fs, with a propagation time step of 4 fs.

With completion of the MD simulations, the programme CREST 2.12^[25, 26]^ was then used, with its CREGEN analysis,^[27]^ to sort the lowest energy conformers (within an energy window of 10 kcal) obtained from the MD simulations into the most favourable geometries (within a RMSD threshold of 0.125). The lowest energy conformer for each complex was then optimised within GFN2-*xTB* 6.7.1^[16]^ with an “extreme” optimization [extreme: Econv (energy convergence) = 5 × 10^−8^ Eh; Gconv (gradient convergence) = 5 × 10^−5^ Eh∙α^−1^; accuracy (for integral cutoffs and SCF criteria) = 0.01] and the solvent DMSO was represented with an implicit solvation model (ALPB, as implemented in the *xTB* code).^[17]^ Lebedev grid level used was “normal” with 230 grid points (as implemented in the *xTB* code).^[18]^ These optimised structures were then used as the input for further optimisation by DFT, again using the PBE0D4 functional with C and H atoms treated by the def2-SVP basis set and all other atoms  (N, O, Pd, Pt) treated by the def2-TZVP basis set.^[23]^

We also optimised the host structures (**[Pd(AD-Bz)_2_]^2+^** and **[Pt(AD-Bz)_2_]^2+^**) and the guests **G_2py_** and **G_2nap_**, using these structures to perform homodesmotic analysis.^[28, 29]^ This allowed us to semi-quantitatively compare each complex by keeping the number and type of atoms/bonds consistent; homodesmotic energetic contributions are thus principally dependent on geometric factors such as ring strain or noncovalent interactions.

For all calculations structures were optimised with tight convergence criteria on both the geometry and self-consistent field (SCF) cycles. The conductor-like polarizable continuum model (CPCM) was used to treat the structures in a DMSO environment.

## 3.2. Electrostatic potential maps

The optimised structures of **[Pt(AD-Bz*_anti_*)_2_]^2+^**, **G_2py_** and **[G_2py_ ⊂ Pt(AD-Bz*_anti_*)_2_]^2+^** created with PBE0D4^[21, 22]^ functionals were used to generate electrostatic potential (ESP) surface maps using Multiwfn.^[30-32]^ The default mapped function is ESP, and the default molecular surface is defined as the isosurface of *ρ* = 0.001. Visuals were constructed using VMD.

**Figure S86** Compared electrostatic potential maps from a side view (left) or top view (right) of a) **[Pt(AD-Bz*_anti_*)_2_]^2+^**, b) **G_2py_** and c) **[G_2py_ ⊂ Pt(AD-Bz*_anti_*)_2_]^2+^**.

## 3.3. Analysis of structures

As discussed, we chose starting geometries that were approximated from solid state data (Figure S105) and predicted by MD simulations (*ie.* approximate solution phase data). These conformers were arranged so that either the guest linker was positioned over the host nitrogen donors with the host solubilising groups *anti* (**[G_2py_ ⊂ M(AD-Bz*_anti_*)_2_]^2+^**, Figure S87a), or the guest linker was positioned over the host aromatic backbone with the host solubilising groups *syn* (**[G_2py_ ⊂ M(AD-Bz*_syn_*)_2_]^2+^**, Figure S87b). To determine whether the presence of the linker on the guests and the host solubilising groups effected the resulting complex energies we also constructed a simplified H-G system, with just two pyrenes and a simplified host (**[pyrene ⊂ M(AD-model)_2_]^2+^**, Figure S87c).

**Figure S87** Example starting geometries (*xTB* optimised structures) of **a)** **[G_2py_ ⊂ Pt(AD-Bz*_anti_*)_2_]^2+^**, **b)** **[G_2py_ ⊂ Pt(AD-Bz*_syn_*)_2_]^2+^**, **c)** **[pyrene ⊂ Pt(AD-model)_2_]^2+^**. The Hydrogen atoms removed for clarity. *NOTE: Only starting geometries of the Pt(II) H-G are shown, for brevity.*

### 3.3.1. Analysis of [G_2_ ⊂ M(AD-Bz*_anti_*)_2_]^2+^ complexes

Complexes analysed at theories PBE and PBE0 showed all guests (**G_2py_**, **G_2nap_** and **G_2Pt_**) moving away from both **[Pd(AD-Bz*_anti_*)_2_]^2+^** and **[Pt(AD-Bz*_anti_*)_2_]^2+^** hosts, though there appeared to be no major difference in dissociation between **[Pd(AD-Bz*_anti_*)_2_]^2+^** and **[Pt(AD-Bz*_anti_*)_2_]^2+^** (Figure S88ai-ii, bi-ii and ci-ii), whilst PBE0D4 showed guests moving closer to the hosts, presumably due to the added dispersion contribution (Figure S88aiii, biii and ciii). Again, despite the guests associating more strongly with the hosts, there appears to be no major differentiation between guest interaction with either **[Pd(AD-Bz*_anti_*)_2_]^2+^** and **[Pt(AD-Bz*_anti_*)_2_]^2+^**. It should be noted that for **G_2Pt_** , the platinum guest centre never seems to sit directly over the **[M(AD-Bz*_anti_*)_2_]^2+^** host centre, indicating that the guest chain is not long enough to allow for full association from both sides of the guest.

**Figure S88** Compared optimised structures of **a)** **[G_2py_ ⊂ M(AD-Bz*_anti_*)_2_]^2+^**, **b)** **[G_2nap_ ⊂ M(AD-Bz*_anti_*)_2_]^2+^**, **c)** **[G_2Pt_ ⊂ M(AD-Bz*_anti_*)_2_]^2+^**, with increasing levels of theory: i) PBE; ii) PBE0; iii) PBE0D4. Dark teal corresponds to **[G_2_ ⊂ Pd(AD-Bz*_anti_*)_2_]^2+^** while white corresponds to **[G_2_ ⊂ Pt(AD-Bz*_anti_*)_2_]^2+^**. Hydrogen atoms removed for clarity.

### 3.3.2. Analysis of [G_2_ ⊂ M(AD-Bz*_syn_*)_2_]^2+^ complexes

Complexes analysed at PBE0D4 showed minimal changes in both distance between the metal centre and guests **G_2py_** and **G_2nap_**. There also appears to be minimal change in guest linker positioning.

**Figure S89** Compared PBE0D4 optimised structures of **a)** **[G_2py_ ⊂ M(AD-Bz*_syn_*)_2_]^2+^** and **b)** **[G_2nap_ ⊂ M(AD-Bz*_syn_*)_2_]^2+^**. Dark teal corresponds to **[G_2_ ⊂ Pd(AD-Bz*_syn_*)_2_]^2+^** while white corresponds to **[G_2_ ⊂ Pt(AD-Bz*_syn_*)_2_]^2+^**. Hydrogen atoms removed for clarity.

### 3.3.3. Analysis of [pyrene ⊂ M(AD-model)_2_]^2+^ complexes

Interestingly, for the simplified complexes calculated at PBE0D4, we saw the most change between complexes **[pyrene ⊂ Pd(AD-model)_2_]^2+^** and **[pyrene ⊂ Pt(AD-model)_2_]^2+^**. For the H-G complex **[pyrene ⊂ Pd(AD-model)_2_]^2+^** we saw a slight shifting off centre for one of the pyrene guests, relative to that observed for **[pyrene ⊂ Pt(AD-model)_2_]^2+^**.

**Figure S90** Compared PBE0D4 optimised structures of **[pyrene ⊂ M(AD-model)_2_]^2+^**. Dark teal corresponds to **[pyrene ⊂ Pd(AD-model)_2_]^2+^** while white corresponds to **[pyrene ⊂ Pt(AD-model)_2_]^2+^**. Hydrogen atoms removed for clarity.

## 3.4. Homodesmotic analysis

### 3.4.1. Analysis of [G_2py_ ⊂ M(AD-Bz*_anti_*)_2_]^2+^ homodesmotic assemblies

**Figure S91** Change in energies of homodesmotic couples **1** (**[G_2py_ ⊂ Pt(AD-Bz*_anti_*)_2_]^2+^∙([Pd(AD-Bz*_anti_*)_2_]^2+^)**) and **2** (**[G_2py_ ⊂ [Pd(AD‑Bz*_anti_*)_2_]^2+^∙([Pt(AD-Bz*_anti_*)_2_]^2+^)**) (PBE0D4 optimised structures). *NOTE: sign is inverse relative to Table S22 to reflect favourability of forming assembly* ***2*** *relative to assembly* ***1.***

**Table S22** Calculated *E*_total_ (E_h_) and *E*_dispersion_ (E_h_) (for PBE0D4) of homodesmotic assemblies **1** and **2**.

| **Homodesmotic component** | *E*_total_ (E_h_) | | | *E*_dispersion_ (E_h_) |
| --- | --- | --- | --- | --- |
|  | PBE | PBE0 | PBE0D4 | PBE0D4 |
| **[Pd(AD-Bz*_anti_*)_2_]^2+^** | -2102.32 | -2102.38 | -2102.48 | -0.098 |
| **[Pt(AD-Bz*_anti_*)_2_]^2+^** | -2093.80 | -2093.85 | -2093.95 | -0.099 |
| **[G_2py_ ⊂ Pd(AD-Bz*_anti_*)_2_]^2+^** | -3866.32 | -3866.53 | -3866.79 | -0.28 |
| **[G_2py_ ⊂ Pt(AD-Bz*_anti_*)_2_]^2+^** | -3857.80 | -3857.99 | -3858.26 | -0.28 |
| **1 – 2** | 0.00060  (1.57 kJ/mol)  (**2** favoured) | -0.00061  (-1.61 kJ/mol)  (**1** favoured) | 0.00031  (0.82 kJ/mol)  (**2** favoured) | 0.00090  (2.36 kJ/mol)  (**2** favoured) |

### 3.4.2. Analysis of [G_2py_ ⊂ M(AD-Bz*_syn_*)_2_]^2+^ homodesmotic assemblies

**Figure S92** Change in energies of homodesmotic couples **3** (**[G_2py_ ⊂ Pt(AD-Bz*_syn_*)_2_]^2+^∙([Pd(AD-Bz*_syn_*)_2_]^2+^)**) and **4** (**[G_2py_ ⊂ [Pd(AD‑Bz*_syn_*)_2_]^2+^∙([Pt(AD-Bz*_syn_*)_2_]^2+^)**) (PBE0D4 optimised structures). *NOTE: sign is inverse relative to Table S23 to reflect favourability of forming assembly* ***4*** *relative to assembly* ***3.***

**Table S23** Calculated *E*_total_ (E_h_) and *E*_dispersion_ (E_h_) (for PBE0D4) of homodesmotic assemblies **3** and **4**.

| **Homodesmotic component** | *E*_total_ (E_h_) | *E*_dispersion_ (E_h_) |
| --- | --- | --- |
|  | PBE0D4 | PBE0D4 |
| **[Pd(AD-Bz*_syn_*)_2_]^2+^** | -2102.48 | -0.099 |
| **[Pt(AD-Bz*_syn_*)_2_]^2+^** | -2093.95 | -0.099 |
| **[G_2py_ ⊂ Pd(AD-Bz*_syn_*)_2_]^2+^** | -3866.81 | -0.28 |
| **[G_2py_ ⊂ Pt(AD-Bz*_syn_*)_2_]^2+^** | -3858.28 | -0.28 |
| **3 – 4** | 0.0022  (5.73 kJ/mol)  (**4** favoured) | 0.00080  (2.08 kJ/mol)  (**4** favoured) |

### 3.4.3. Analysis of [G_2nap_ ⊂ M(AD-Bz*_anti_*)_2_]^2+^ homodesmotic assemblies

**Figure S93** Change in energies of homodesmotic couples **5** (**[G_2nap_ ⊂ Pt(AD-Bz*_anti_*)_2_]^2+^∙([Pd(AD-Bz*_anti_*)_2_]^2+^)**) and **6** (**[G_2nap_ ⊂ [Pd(AD‑Bz*_anti_*)_2_]^2+^∙([Pt(AD-Bz*_anti_*)_2_]^2+^)**). *NOTE: sign is inverse relative to Table S24 to reflect favourability of forming assembly* ***6*** *relative to assembly* ***5.***

**Table S24** Calculated *E*_total_ (E_h_) and *E*_dispersion_ (E_h_) (for PBE0D4) of homodesmotic assemblies **5** and **6**.

| **Homodesmotic component** | *E*_total_ (E_h_) | | | | *E*_dispersion_ (E_h_) |
| --- | --- | --- | --- | --- | --- |
|  | PBE | PBE0 | PBE0D4 | PBE0D4 | |
| **[Pd(AD-Bz*_anti_*)_2_]^2+^** | -2102.32 | -2102.38 | -2102.48 | -0.098 | |
| **[Pt(AD-Bz*_anti_*)_2_]^2+^** | -2093.80 | -2093.85 | -2093.95 | -0.099 | |
| **[G_2nap_ ⊂ Pd(AD-Bz*_anti_*)_2_]^2+^** | -3407.44 | -3407.62 | -3407.83 | -0.23 | |
| **[G_2nap_ ⊂ Pt(AD-Bz*_anti_*)_2_]^2+^** | -3398.93 | -3399.08 | -3399.30 | -0.22 | |
| **5 – 6** | 0.00056  (1.47 kJ/mol)  (**6** favoured) | -0.0012  (-3.24 kJ/mol)  (**5** favoured) | 0.0036  (9.46 kJ/mol)  (**6** favoured) | 0.0046  (12.0 kJ/mol)  (**6** favoured) | |

### 3.4.4. Analysis of [G_2nap_ ⊂ M(AD-Bz*_syn_*)_2_]^2+^ homodesmotic assemblies

**Figure S94** Change in energies of homodesmotic couples **7** (**[G_2nap_ ⊂ Pt(AD-Bz*_syn_*)_2_]^2+^∙([Pd(AD-Bz*_syn_*)_2_]^2+^)**) and **8** (**[G_2nap_ ⊂ [Pd(AD‑Bz*_syn_*)_2_]^2+^∙([Pt(AD-Bz*_syn_*)_2_]^2+^)**). *NOTE: sign is inverse relative to Table S25 to reflect favourability of forming assembly* ***8*** *relative to assembly* ***7.***

**Table S25** Calculated *E*_total_ (E_h_) and *E*_dispersion_ (E_h_) (for PBE0D4) of homodesmotic assemblies **7** and **8**.

| **Homodesmotic component** | *E*_total_ (E_h_) | *E*_dispersion_ (E_h_) |
| --- | --- | --- |
|  | PBE0D4 | PBE0D4 |
| **[Pd(AD-Bz*_syn_*)_2_]^2+^** | -2102.48 | -0.099 |
| **[Pt(AD-Bz*_syn_*)_2_]^2+^** | -2093.95 | -0.099 |
| **[G_2nap_ ⊂ Pd(AD-Bz*_syn_*)_2_]^2+^** | -3407.84 | -0.23 |
| **[G_2nap_ ⊂ Pt(AD-Bz*_syn_*)_2_]^2+^** | -3399.31 | -0.23 |
| **7 – 8** | 0.0012  (3.28 kJ/mol)  (**8** favoured) | 0.00028  (0.73 kJ/mol)  (**8** favoured) |

### 3.4.5. Analysis of [G_2Pt_ ⊂ M(AD-Bz*_anti_*)_2_]^2+^ homodesmotic assemblies

**Figure S95** Change in energies of homodesmotic couples **9** (**[G_2Pt_ ⊂ Pt(AD-Bz*_anti_*)_2_]^2+^∙([Pd(AD-Bz*_anti_*)_2_]^2+^)**) and **10** (**[G_2Pt_ ⊂ [Pd(AD‑Bz*_anti_*)_2_]^2+^∙([Pt(AD-Bz*_anti_*)_2_]^2+^)**). *NOTE: sign is inverse relative to Table S26 to reflect favourability of forming assembly* ***9*** *relative to assembly* ***10.***

**Table S26** Calculated *E*_total_ (E_h_) and *E*_dispersion_ (E_h_) (for PBE0D4) of homodesmotic assemblies **9** and **10**.

| **Homodesmotic component** | *E*_total_ (E_h_) | | | | *E*_dispersion_ (E_h_) |
| --- | --- | --- | --- | --- | --- |
|  | PBE | PBE0 | PBE0D4 | PBE0D4 | |
| **[Pd(AD-Bz*_anti_*)_2_]^2+^** | -2102.32 | -2102.38 | -2102.48 | -0.098 | |
| **[Pt(AD-Bz*_anti_*)_2_]^2+^** | -2093.80 | -2093.85 | -2093.95 | -0.099 | |
| **[G_2Pt_ ⊂ Pd(AD-Bz*_anti_*)_2_]^2+^** | -4176.82 | -4176.69 | -4177.18 | -0.29 | |
| **[G_2Pt_ ⊂ Pt(AD-Bz*_anti_*)_2_]^2+^** | -4168.30 | -4168.16 | -4168.65 | -0.30 | |
| **9 – 10** | 0.0011  (2.80 kJ/mol)  (**10** favoured) | 0.0014  (3.67 kJ/mol)  (**10** favoured) | -0.00012  (-0.31 kJ/mol)  (**9** favoured) | -0.00074  (-1.95 kJ/mol)  (**9** favoured) | |

### 3.4.6. Analysis of [pyrene ⊂ M(AD-model)_2_]^2+^ homodesmotic assemblies

**Figure S96** Change in energies of homodesmotic couples **11** (**[pyrene ⊂ Pt(AD-model)_2_]^2+^∙([Pd(AD-model)_2_]^2+^)**) and **12** (**[pyrene ⊂ [Pd(AD‑model)_2_]^2+^∙([Pt(AD-model)_2_]^2+^)**). *NOTE: AD-model represents the simplified AD-Bz ligand used for these studies in which the solubilising chain has been removed; sign is inverse relative to Table S27 to reflect favourability of forming assembly* ***11*** *relative to assembly* ***12.***

**Table S27** Calculated *E*_total_ (E_h_) and *E*_dispersion_ (E_h_) (for PBE0D4) of homodesmotic assemblies **11** and **12**.

| **Homodesmotic component** | *E*_total_ (E_h_) | *E*_dispersion_ (E_h_) |
| --- | --- | --- |
|  | PBE0D4 | PBE0D4 |
| **[Pd(AD-model)_2_]^2+^** | -1105.10 | -0.048 |
| **[Pt(AD-model)_2_]^2+^** | -1096.57 | -0.049 |
| **[pyrene ⊂ Pd(AD-model)_2_]^2+^** | -2334.49 | -0.18 |
| **[pyrene ⊂ Pt(AD-model)_2_]^2+^** | -2325.96 | -0.19 |
| **11 – 12** | -0.0032  (-8.45 kJ/mol)  (**11** favoured) | -0.0036  (-9.41 kJ/mol)  (**11** favoured) |

## 3.5. Molecular dynamics simulations

Also already mentioned, molecular dynamic (MD) simulations were employed in order to probe the dynamic equilibrium of the complexes **[G_2py_ ⊂ Pd(AD-Bz)_2_]^2+^**, **[G_2py_ ⊂ Pt(AD-Bz)_2_]^2+^**, **[G_2nap_ ⊂ Pd(AD-Bz)_2_]^2+^** and **[G_2nap_ ⊂ Pt(AD‑Bz)_2_]^2+^**.

To analyse the resulting simulation trajectories, relative change in energy (Δ*E*) was used to assess the changes in the complex structure, though only energetic changes relating to the ‘sliding’ of the guest over the square planar host were observed, with the distance between guest and host remaining effectively constant. For this analysis, the first 90 frames were removed from the data set so as to remove the initial loose optimization and ensure that the system was at equilibrium.

### 3.5.1. Dynamic equilibrium of [G_2py_ ⊂ Pd(AD-Bz)_2_]^2+^

The MD simulation of **[G_2py_ ⊂ Pd(AD-Bz)_2_]^2+^**, initially places the guest wrapped around the square planar host with pyrene motifs close in space to either side of the host. The distance between the pyrene motifs and host remains close and fairly constant. The panels slide across the face of the host loosely before settling with the tether of **G_2py_** shifting positions (Figure S97), accounting for a small initial decrease in energy from ~20-50 ps. Beyond this point minimal changes were observed, reflected by the overall consistent energy of the system (Figure S98).

**Figure S97** Comparison of the initial geometry of **[G_2py_ ⊂ Pd(AD-Bz)_2_]^2+^** of the MD simulation, depicting the shift in positioning of the tether of **G_2py_** at a) 20 ps, and b) 50 ps.

 

**Figure S98** MD simulation time trace of **[G_2py_ ⊂ Pd(AD-Bz)_2_]^2+^**, showing relative change in energy (Δ*E*_rel_), with pale data showing full energy change, while bold lines show rolling average (intervals of 25 ps).

### 3.5.2. Dynamic equilibrium of [G_2py_ ⊂ Pt(AD-Bz)_2_]^2+^

The MD simulation of **[G_2py_ ⊂ Pt(AD-Bz)_2_]^2+^**, initially places the guest wrapped around the square planar host with pyrene motifs close in space to either side of the host. The distance between the pyrene motifs and host remains close and fairly constant, with minimal changes observed, reflected by the overall consistent energy of the system (Figure S99).

 

**Figure S99** MD simulation time trace of  **[G_2py_ ⊂ Pt(AD-Bz)_2_]^2+^**, showing relative change in energy (Δ*E*_rel_), with pale data showing full energy change, while bold lines show rolling average (intervals of 25 ps).

### 3.5.3. Dynamic equilibrium of [G_2nap_ ⊂ Pd(AD-Bz)_2_]^2+^

The MD simulation of **[G_2nap_ ⊂ Pd(AD-Bz)_2_]^2+^**, initially places the guest wrapped around the square planar host with naphthalene motifs close in space to either side of the host. The distance between the naphthalene motifs and host remains close and fairly constant, with minimal changes observed, reflected by the overall consistent energy of the system (Figure S100).

**Figure S100** MD simulation time trace of **[G_2nap_ ⊂ Pd(AD-Bz)_2_]^2+^**, showing relative change in energy (Δ*E*_rel_), with pale data showing full energy change, while bold lines show rolling average (intervals of 25 ps).

### 3.5.4. Dynamic equilibrium of [G_2nap_ ⊂ Pt(AD-Bz)_2_]^2+^

The MD simulation of **[G_2nap_ ⊂ Pt(AD-Bz)_2_]^2+^**, initially places the guest wrapped around the square planar host with naphthalene motifs close in space to either side of the host. The distance between the naphthalene motifs and host remains close and fairly constant. The panels slide across the face of the host loosely before settling with naphthalene motifs stacked offset but near the Pd(II) metal centre (Figure S101), accounting for a small initial decrease in energy from ~320-350 ps. Beyond this point minimal changes were observed, reflected by the overall consistent energy of the system (Figure S102).

**Figure S101** Comparison of the initial geometry of **[G_2nap_ ⊂ Pt(AD-Bz)_2_]^2+^** in the MD simulation, depicting the rearrangement of **G_2nap_** at a) 320 ps and b) 350 ps.

**Figure S102** MD simulation time trace of **[G_2nap_ ⊂ Pt(AD-Bz)_2_]^2+^**, showing relative change in energy (Δ*E*_rel_), with pale data showing full energy change, while bold lines show rolling average (intervals of 25 ps).

# 4. Crystallography

## 4.1. Experimental data

Single crystal data were collected using mirror-monochromated Cu Kα radiation on an Agilent SuperNova diffractometer at 150 K. Raw frame data (including data reduction, interframe scaling, unit cell refinement and absorption corrections) were processed using CrysAlis Pro.^[33]^ All structures were solved by direct methods using SHELXT2014^[34, 35]^ and refined against all F2 data using SHELXL2014^[35]^ and the OLEX2^[36]^ interface. The data are summarised in Table S28. A CheckCIF report was generated for each structure, and responses to the A-B level alerts are provided in the CIFs.

Despite several attempts, crystals of the host with tethered guests, **G_2py_**, **G_2nap_** or **G_2Pt_** bound were not obtained. From the crystal structures of the hosts, **[Pd(AD-Bz)_2_](BF_4_)_2_** and **[Pt(AD-Bz)_2_](BF_4_)_2_**, the metal centres pack with the aromatic solubilising chains, likely preventing co-crystallisation of the host with the guests. To combat this, a previously synthesised host, **[Pd(AD-PEG)_2_](BF_4_)_2_**,^[2]^ and similar, novel host, **[Pt(AD-PEG)_2_](BF_4_)_2_**, was used with **G_py_** and **G_Pt_**.^[3]^

Table S28 Summary of crystallographic data.

|  | **[Pd(AD-Bz)_2_](BF_4_)_2_** | **[Pt(AD-Bz)_2_](BF_4_)_2_** | **[G_py_** ⊂ **Pd(AD-PEG)_2_](BF_4_)_2_** |
| --- | --- | --- | --- |
| **CCDC No.** | **2564264** | **2564265** | **2564266** |
| Formula | C_32_H_32_N_8_O_4_PdB_2_F_8_ | C_32_H_32_N_8_O_4_PtB_2_F_8_ | C_31_H_35_N_8_O_4_PdB_2_F_8_ |
| *M* | 872.67 | 961.36 | 863.69 |
| *T* (K) | 150 | 150 | 150 |
| Crystal system | Monoclinic | Monoclinic | Orthorhombic |
| Space group | *P* 1 2_1_/*n* 1 | *P* 1 2_1_/*n* 1 | Pbcn |
| *a* (Å) | 8.59090(10) | 8.57670(10) | 19.3087(4) |
| *b* (Å) | 15.39830(10) | 15.4412(2) | 18.8101(4) |
| *c* (Å) | 13.29040(10) | 13.27530(10) | 20.2150(4) |
| *α* (°) | 90 | 90 | 90 |
| *β* (°) | 99.7330(10) | 99.0630(10) | 90 |
| *γ* (°) | 90 | 90 | 90 |
| *V* (Å^3^) | 1732.82(3) | 1736.16(3) | 7342.1(3) |
| *Z* [*Z’*] | 2 [0.5] | 8 [0.5] | 8 [1] |
| Crystal description | Colourless block | Colourless block | Orange needle |
| Crystal size (mm^3^) | 0.2 × 0.2 × 0.1 | 0.2 × 0.2 × 0.1 | 0.3 × 0.05 × 0.04 |
| *μ* (mm^–1^) | 5.167 | 8.405 | 4.849 |
| 2*θ*_full_, 2*θ*_max_ (°) | 135.37, 156.12 | 135.37, 151.97 | 135.37, 152.32 |
| *N*_measured refl_ | 9854 | 10024 | 25856 |
| *N*_independent refl_ [*R*_int_] | 8814 [0.0194] | 8618 [0.0799] | 7853 [0.0443] |
| *N*_observed refl_ [*I* > 2*σ*(*I*)] | 3076 | 3294 | 5122 |
| *N*_parameters_ | 252 | 252 | 399 |
| *N*_restraints_ | 0 | 0 | 73 |
| *R* [*I* > 2*σ*(*I*)] | 0.0311 | 0.0865 | 0.0782 |
| *wR* [all data] | 0.0833 | 0.02063 | 0.2413 |
| GOF | 1.072 | 1.129 | 1.040 |
| ∆*ρ*_max_, ∆*ρ*_min_ (e Å^-3^) | 0.914, -0.473 | 3.531, -2.830 | 2.271, -0.830 |
| Solvent mask* | N/A | N/A | 2168 A^3^, 656 e^-^ |

*Solvent mask implemented with OLEX2 package.^[36]^

Table S28 (*cont.*) Summary of crystallographic data.

|  | **[G_Pt_** ⊂ **Pd(AD-PEG)_2_](BF_4_)_2_** |  |  | **[G_Pt_** ⊂ **Pt(AD-PEG)_2_](BF_4_)_2_** |
| --- | --- | --- | --- | --- |
| **CCDC No.** | **2564267** |  |  | **2564268** |
| Formula | C_37_H_45_N_9_O_6_PdPtB_2_F_8_S_2_ |  |  | C_37_H_45_N_9_O_6_Pt_2_B_2_F_8_S_2_ |
| *M* | 1251.05 |  |  | 1339.74 |
| *T* (K) | 150 |  |  | 150 |
| Crystal system | Monoclinic |  |  | Monoclinic |
| Space group | P 1 2_1_ 1 |  |  | P 1 2_1_ 1 |
| *a* (Å) | 7.09170(10) |  |  | 7.09620(10) |
| *b* (Å) | 22.7908(2) |  |  | 22.7668(3) |
| *c* (Å) | 14.13390(10) |  |  | 14.1461(2) |
| *α* (°) | 90 |  |  | 90 |
| *β* (°) | 102.3790(10) |  |  | 102.420(2) |
| *γ* (°) | 90 |  |  | 90 |
| *V* (Å^3^) | 2231.29(4) |  |  | 2231.93(6) |
| *Z* [*Z’*] | 2 [1] |  |  | 2 [1] |
| Crystal description | Orange needle |  |  | Yellow needle |
| Crystal size (mm^3^) | 0.2 × 0.09 × 0.05 |  |  | 0.3 × 0.04 × 0.02 |
| *μ* (mm^–1^) | 10.697 |  |  | 13.221 |
| 2*θ*_full_, 2*θ*_max_ (°) | 135.37, 156.86 |  |  | 135.37, 155.99 |
| *N*_measured refl_ | 15328 |  |  | 11833 |
| *N*_independent refl_ [*R*_int_] | 14098 [0.0180] |  |  | 9631 [0.0476] |
| *N*_observed refl_ [*I* > 2*σ*(*I*)] | 7669 |  |  | 6421 |
| *N*_parameters_ | 620 |  |  | 625 |
| *N*_restraints_ | 55 |  |  | 31 |
| *R* [*I* > 2*σ*(*I*)] | 0.0297 |  |  | 0.0464 |
| *wR* [all data] | 0.0758 |  |  | 0.1224 |
| GOF | 1.042 |  |  | 1.030 |
| ∆*ρ*_max_, ∆*ρ*_min_ (e Å^-3^) | 1.784, -2.120 |  |  | 1.748, -1.796 |
| Solvent mask | N/A |  |  | N/A |

1.1. **ORTEP diagrams**

Structure of **[Pd(AD-Bz)_2_](BF_4_)_2_** : the main molecule and counterions were well behaved and required no restraints.

Figure S103 ORTEP diagram of the molecular unit of [Pd(AD-Bz)_2_](BF_4_)_2_. Ellipsoids are shown to the 50% probability level.

Structure of **[Pt(AD-Bz)_2_](BF_4_)_2_**: the main molecule and counterions were well behaved and required no restraints.

Figure S104 ORTEP diagram of the molecular unit of [Pd(AD-Bz)_2_](BF_4_)_2_. Ellipsoids are shown to the 50% probability level.

Structure of **[G_py_** ⊂ **Pd(AD-PEG)_2_](BF_4_)_2_**: the main molecule was generally well behaved, but the position and ADPs of many atoms associated with solubilising chains, **G_py_** and counterions required restraints. **G_py_** was likely disordered across two orientations which resulted in significant disorder in the solubilising chain of the guest. This solubilising chain and one significantly disordered BF_4_ counterion were accounted for by use of a solvent mask, implemented within the OLEX2 package.

Figure S105 ORTEP diagram of the molecular unit of [G_py_ ⊂ Pd(AD-PEG)_2_](BF_4_)_2_. Ellipsoids are shown to the 50% probability level. Solvent mask was used in refinement, implemented within the OLEX2 package.

Structure of **[G_Pt_** ⊂ **Pd(AD-PEG)_2_](BF_4_)_2_**: the main molecule, solvent and counterions were generally well behaved. Restraints were only required at the imine bond of **G_Pt_** which was likely disordered across two orientations.

Figure S106 ORTEP diagram of the molecular unit of [G_Pt_ ⊂ Pd(AD-PEG)_2_](BF_4_)_2_. Ellipsoids are shown to the 50% probability level.

Structure of **[G_Pt_** ⊂ **Pt(AD-PEG)_2_](BF_4_)_2_**: the main molecule, solvent and counterions were generally well behaved. Restraints were only required at the imine bond of **G_Pt_** which was likely disordered across two orientations, and a single disordered BF_4_.

Figure S107 ORTEP diagram of the molecular unit of [G_Pt_ ⊂ Pt(AD-PEG)_2_](BF_4_)_2_. Ellipsoids are shown to the 50% probability level.

# 2. References

[1]	F. Moradgholi, H. Vahedi, J. Lari, "Simple and novel synthetic method to mixed-donor podand" *Organic Communications* ***8****,* (2015): 52-59.

[2]	J. L. Algar, J. E. Phillips, J. D. Evans, D. Preston, "Stoichiometric Control of Guest Recognition of Self-Assembled Palladium(II)-Based Supramolecular Architectures" *Chemistry - An Asian Journal* ***18****,* (2023): e202300673.

[3]	H. Motschi, C. Nussbaumer, P. S. Pregosin, F. Bachechi, P. Mura, L. Zambonelli, "The trans-Influence in Platinum (II) Complexes. 1H- and 13C-NMR. and X-ray structural studies of tridentate Schiff's base complexes of platinum (II)" *Helvetica Chimica Acta* ***63****,* (1980): 2071-2086.

[4]	K. Stott, J. Stonehouse, J. Keeler, T.-L. Hwang, A. J. Shaka, "Excitation Sculpting in High-Resolution Nuclear Magnetic Resonance Spectroscopy: Application to Selective NOE Experiments" *Journal of the American Chemical Society* ***117****,* (1995): 4199-4200.

[5]	J. Jeneer, B. Meier, P. Bachmann, R. Ernst, "Investigation of exchange processes by two-dimensional NMR spectroscopy" *The Journal of Chemical Physics* ***71****,* (1979): 4546-4553.

[6]	R. Wagner, S. Berger, "Gradient-Selected NOESY-A Fourfold Reduction of the Measurement Time for the NOESY Experiment" *Journal of Magnetic Resonance, Series A* ***123****,* (1996): 119-121.

[7]	A. Jerschow, N. Müller, "3D Diffusion-Ordered TOCSY for Slowly Diffusing Molecules" *Journal of Magnetic Resonance, Series A* ***123****,* (1996): 222-225.

[8]	A. Jerschow, N. Müller, "Suppression of convection artifacts in stimulated-echo diffusion experiments. Double-stimulated-echo experiments" *Journal of Magnetic Resonance* ***125****,* (1997): 372-375.

[9]	J. Kolien, A. R. Inglis, R. A. S. Vasdev, B. I. Howard, P. E. Kruger, D. Preston, "Exploiting the labile site in dinuclear [Pd2L2]n+ metallo-cycles: multi-step control over binding affinity without alteration of core host structure" *Inorganic Chemistry Frontiers* ***7****,* (2020): 3895-3908.

[10]	P. Thordarson, J. Wilmot, V. Efremova, *Bindfit.* <http://supramolecular.org>.

[11]	P. Thordarson, "Determining association constants from titration experiments in supramolecular chemistry" *Chemical Society Reviews* ***40****,* (2011): 1305-1323.

[12]	D. Brynn Hibbert, P. Thordarson, "The death of the Job plot, transparency, open science and online tools, uncertainty estimation methods and other developments in supramolecular chemistry data analysis" *Chemical Communications* ***52****,* (2016): 12792-12805.

[13]	A beta version of this software is available at: <https://github.com/martinp23/bindmc/releases>.

[14]	D. O. Soloviev, C. A. Hunter, "Musketeer: a software tool for the analysis of titration data" *Chemical Science* ***15****,* (2024): 15299-15310.

[15]	Wavefunction Inc., **2025**.

[16]	C. Bannwarth, S. Ehlert, S. Grimme, "GFN2-xTB-An Accurate and Broadly Parametrized Self-Consistent Tight-Binding Quantum Chemical Method with Multipole Electrostatics and Density-Dependent Dispersion Contributions" *Journal of Chemical Theory and Computation* ***15****,* (2019): 1652-1671.

[17]	G. Sigalov, A. Fenley, A. Onufriev, "Analytical electrostatics for biomolecules: beyond the generalized Born approximation" *The Journal of Chemical Physics* ***124****,* (2006): 124902.

[18]	V. I. Lebedev, "Spherical quadrature formulas exact to orders 25–29" *Siberian Mathematical Journal* ***18****,* (1977): 99-107.

[19]	F. Neese, "Software Update: The ORCA Program System—Version 6.0" *WIREs Computational Molecular Science* ***15****,* (2025): e70019.

[20]	M. Ropo, K. Kokko, L. Vitos, "Assessing the Perdew-Burke-Ernzerhof exchange-correlation density functional revised for metallic bulk and surface systems" *Physical Review B* ***77****,* (2008): 195445.

[21]	C. Adamo, V. Barone, "Toward reliable density functional methods without adjustable parameters: The PBE0 model" *The Journal of Chemical Physics* ***110****,* (1999): 6158-6170.

[22]	E. Caldeweyher, S. Ehlert, A. Hansen, H. Neugebauer, S. Spicher, C. Bannwarth, S. Grimme, "A generally applicable atomic-charge dependent London dispersion correction" *The Journal of Chemical Physics* ***150****,* (2019).

[23]	F. Weigend, R. Ahlrichs, "Balanced basis sets of split valence, triple zeta valence and quadruple zeta valence quality for H to Rn: Design and assessment of accuracy" *Physical Chemistry Chemical Physics* ***7****,* (2005): 3297-3305.

[24]	J.-P. Ryckaert, G. Ciccotti, H. J. C. Berendsen, "Numerical integration of the cartesian equations of motion of a system with constraints: molecular dynamics of n-alkanes" *Journal of Computational Physics* ***23****,* (1977): 327-341.

[25]	S. Grimme, "Exploration of Chemical Compound, Conformer, and Reaction Space with Meta-Dynamics Simulations Based on Tight-Binding Quantum Chemical Calculations" *J Chem Theory Comput* ***15****,* (2019): 2847-2862.

[26]	P. Pracht, F. Bohle, S. Grimme, "Automated exploration of the low-energy chemical space with fast quantum chemical methods" *Phys Chem Chem Phys* ***22****,* (2020): 7169-7192.

[27]	P. Pracht, S. Grimme, C. Bannwarth, F. Bohle, S. Ehlert, G. Feldmann, J. Gorges, M. Muller, T. Neudecker, C. Plett, S. Spicher, P. Steinbach, P. A. Wesolowski, F. Zeller, "CREST-A program for the exploration of low-energy molecular chemical space" *J Chem Phys* ***160****,* (2024).

[28]	P. George, M. Trachtman, C. W. Bock, A. M. Brett, "An alternative approach to the problem of assessing stabilization energies in cyclic conjugated hydrocarbons" *Theoretica chimica acta* ***38****,* (1975): 121-129.

[29]	P. George, M. Trachtman, C. W. Bock, A. M. Brett, "Homodesmotic reactions for the assessment of stabilization energies in benzenoid and other conjugated cyclic hydrocarbons" *Journal of the Chemical Society, Perkin Transactions 2* (1976): 1222-1227.

[30]	T. Lu, F. Chen, "Multiwfn: a multifunctional wavefunction analyzer" *Journal of Computational Chemistry* ***33****,* (2012): 580-592.

[31]	T. Lu, "A comprehensive electron wavefunction analysis toolbox for chemists, Multiwfn" *The Journal of Chemical Physics* ***161****,* (2024).

[32]	J. Zhang, T. Lu, "Efficient evaluation of electrostatic potential with computerized optimized code" *Physical Chemistry Chemical Physics* ***23****,* (2021): 20323-20328.

[33]	Agilent Technologies, Yarnton, Oxfordshire, UK, **2019**.

[34]	G. M. Sheldrick, "A short history of SHELX" *Acta Crystallographica Section A* ***64****,* (2008): 112-122.

[35]	G. M. Sheldrick, "SHELXT - integrated space-group and crystal-structure determination" *Acta Crystallographica Section A: Foundations and Advances* ***71****,* (2015): 3-8.

[36]	O. V. Dolomanov, L. J. Bourhis, R. J. Gildea, J. A. K. Howard, H. Puschmann, "OLEX2: a complete structure solution, refinement and analysis program" *Journal of Applied Crystallography* ***42****,* (2009): 339-341.
